# Wild Flora Species from Romania with Anxiolytic and Antidepressant Potential: A Global Perspective—Narrative Review

**DOI:** 10.3390/biomedicines14051019

**Published:** 2026-04-30

**Authors:** Olimpia-Daniela Frenț, Eleonora Marian, Laura Grațiela Vicaș, Ioana Lavinia Dejeu, George Emanuiel Dejeu, Mariana Ganea, Georgiana Ioana Potra Cicalău, Gabriela Ciavoi, Roxana Alexandra Cristea, Csaba Nagy, Darius Aghaei, Claudiu-Sorin Iova

**Affiliations:** 1 Department of Pharmacy, Faculty of Medicine and Pharmacy, University of Oradea, No. 29 Nicolae Jiga Street, 410028 Oradea, Romania; ofrent@uoradea.ro (O.-D.F.); emarian@uoradea.ro (E.M.); ioana.dejeu@csud.uoradea.ro (I.L.D.); 2 Department of Surgical Disciplines, Faculty of Medicine and Pharmacy, University of Oradea, 10 Piata 1 Decembrie Street, 410073 Oradea, Romania; dejeu.george@uoradea.ro; 3 Department of Dental Medicine, Faculty of Medicine and Pharmacy, University of Oradea, 10 1st Decembrie Street, 410073 Oradea, Romania; cicalau.georgiana@uoradea.ro (G.I.P.C.); gciavoi@uoradea.ro (G.C.); 4 Doctoral School of Biomedical Sciences, University of Oradea, 1 Universitătii Street, 410073 Oradea, Romania; cristea.roxana.alexandra@didactic.uoradea.ro (R.A.C.); nagy.csaba@student.uoradea.ro (C.N.); aghaei.darius@student.uoradea.ro (D.A.); 5 Clinical Hospital of Neurology and Psychiatry, Louis Pasteur Street, 410154 Oradea, Romania; sorin.iova@didactic.uoradea.ro; 6 Department of Psycho-Neurosciences and Rehabilitation, Faculty of Medicine and Pharmacy, University of Oradea, 410087 Oradea, Romania

**Keywords:** depression, anxiety, phytotherapy, medicinal plants, antioxidant activity, phytochemicals

## Abstract

**Introduction:** Depression and anxiety are highly prevalent disorders with a substantial impact on quality of life. Limitations related to the efficacy and tolerability of conventional pharmacological treatments have stimulated increasing interest in complementary therapeutic approaches, including phytotherapy. This review aims to provide an integrative analysis of some plant species present in the spontaneous flora of Romania, correlating their traditional use with the phytochemical, pharmacological, preclinical, and clinical data available globally. The approach aims to highlight the therapeutic relevance of these species in both regional and international contexts. **Relevant sections:** This narrative review integrates available data on seven species commonly used in traditional medicine: *Matricaria chamomilla* L., *Galium odoratum* L., *Melissa officinalis* L., *Leonurus cardiaca* L., *Hypericum perforatum* L., *Tilia* spp., and *Crataegus monogyna* Jacq. This review examines their geographical distribution, taxonomic classification, phytochemical composition, proposed mechanisms of action, and available preclinical and clinical evidence, as well as safety considerations and products currently available on the Romanian pharmaceutical sales. **Discussion:** Current evidence suggests that *Hypericum perforatum* L. and *Melissa officinalis* L. are supported by relatively robust clinical data regarding their efficacy in reducing anxiety and depressive symptoms. For the remaining species, evidence is derived mainly from preclinical studies or traditional use. The proposed mechanisms of action include modulation of neurotransmitter activity, antioxidant and anti-inflammatory effects, and regulation of the hypothalamic–pituitary–adrenal (HPA) axis. **Conclusions:** Phytotherapy represents a promising approach in the management of anxiety and depressive disorders, particularly as a complementary therapeutic option. However, the strength of evidence varies considerably among the analyzed species, and clinical data remain limited for several of them. **Future directions:** From a future perspective, advancing the clinical relevance of the analyzed plant species requires a more coherent integration of existing pharmacological, preclinical, and emerging clinical data. Particular attention should be given to species for which the current evidence remains predominantly experimental, by promoting research strategies that facilitate the translation of mechanistic findings into clinically meaningful outcomes.

## 1. Introduction

Depression and anxiety disorders represent major global public health concerns, characterized by high prevalence and a profound impact on psychosocial functioning and quality of life. According to data from the World Health Organization, depression affects more than 280 million individuals worldwide, while anxiety disorders account for approximately 260 million cases, highlighting the magnitude and clinical relevance of these conditions [1].

Although global data indicate a high prevalence of anxiety and depression, available epidemiological estimates for Romania suggest lower values. Thus, in 2019, the rate of mental disorders was approximately 11,423 cases per 100,000 inhabitants, of which 3368 were attributed to depressive disorders and 3711 to anxiety disorders. These values are comparable to the average recorded in Central Europe, but remain lower than those reported globally and significantly lower than those in Western Europe [2]. More recent studies, conducted on specific populations, however, indicate considerably higher values, such as one study reporting that 34.0% of Romanian students at the Faculty of Medicine in Galați presented significant clinical depressive symptoms, with a higher frequency among females [3]. These results suggest the existence of important differences between data reported at the population level and those observed in specific groups, possibly influenced by socio-demographic and contextual factors.

Although conventional pharmacological treatment—primarily based on antidepressants and anxiolytics such as selective serotonin reuptake inhibitors (SSRIs), serotonin–norepinephrine reuptake inhibitors (SNRIs), tricyclic antidepressants (TCAs), and monoamine oxidase inhibitors (MAOIs)—is widely used, it is frequently associated with significant adverse effects and challenges related to tolerability and treatment adherence. Commonly reported side effects include gastrointestinal disturbances, tachycardia, dry mouth, orthostatic hypotension, urinary retention, visual disturbances, insomnia, and discontinuation-related symptoms [4,5].

These limitations have contributed to growing interest in alternative or complementary therapeutic approaches, including phytotherapy. Herbal medicine is widely employed as a complementary or alternative strategy for managing depressive and anxiety symptoms, as numerous plant-based preparations—available as dietary supplements or over-the-counter (OTC) medicinal products—are perceived to have a more favorable safety profile and to cause fewer adverse effects compared with conventional pharmacotherapy [4,6]. This trend is further supported by World Health Organization reports indicating that over 80% of the population in developing countries, particularly in rural areas, relies on natural products as a primary source of healthcare [5,7].

The use of medicinal plants in the management of anxiety–depressive symptoms is widespread both internationally and in Romania, where numerous wild-growing species have traditionally been employed for this purpose. However, despite this diversity, only a limited number of species have been sufficiently investigated and supported by pharmacological or clinical evidence regarding their anxiolytic and antidepressant effects. Although herbal preparations are often perceived as safer than synthetic drugs, the level of scientific evidence supporting their efficacy and safety remains variable. Preclinical studies have demonstrated antidepressant and anxiolytic activities for many plant species, mediated through mechanisms such as modulation of neurotransmitter pathways, anti-inflammatory effects, regulation of the hypothalamic–pituitary–adrenal (HPA) axis, and enhancement of neuroplasticity. However, clinical data remain insufficient across different species, reflecting differences in study design, extract standardization, and experimental models [8,9].

In Romania, plant species commonly used in the phytotherapeutic management of anxiety and depression include chamomile (*Matricaria chamomilla* L.), sweet woodruff (*Galium odoratum* (L.) Scop.), lemon balm (*Melissa officinalis* L.), motherwort (*Leonurus cardiaca* L.), St. John’s wort (*Hypericum perforatum* L.), linden (*Tilia* spp.), and hawthorn (*Crataegus monogyna* Jacq.). These species are recognized for their rich content of bioactive compounds, including flavonoids, phenolic acids, naphthodianthrones, phloroglucinols, and proanthocyanidins; alkaloids such as leonurine [10], iridoids [11], and sesquiterpenes [12]; and other constituents with potential neuroprotective, anxiolytic, and antidepressant properties [6].

Romania’s biodiversity, characterized by a rich and relatively well-preserved spontaneous flora at the European level, which includes numerous native species that develop in relatively little-altered natural habitats, represents an important resource for the identification and investigation of bioactive compounds with potential application in modern phytotherapy. This floristic diversity provides a favorable framework for the systematic exploration of traditionally used medicinal plants and for their evaluation in light of current scientific evidence.

This review aims to provide an integrative analysis of plant species present in Romania’s spontaneous flora, correlating their traditional use with phytochemical, pharmacological, preclinical, and clinical data available globally. The approach aims to highlight the therapeutic relevance of these species in both regional and international contexts.

Although the analyzed plant species are widely distributed and used globally, this review focuses on their presence in the spontaneous flora of Romania and on their traditional use in this specific ethnopharmacological context. The aim is not to limit the relevance of these species to the regional level but to provide a geographically anchored perspective, integrated with the scientific evidence available internationally.

From a pharmacological point of view, these plants are of particular interest due to their effects on the central nervous system, as they are traditionally used and scientifically validated for their anxiolytic and antidepressant properties. Thus, *Hypericum perforatum* L. contains active compounds such as hypericin and hyperforin, involved in the modulation of neurotransmitters (especially serotonin), conferring a clinically proven antidepressant effect. The other species, such as *Melissa officinalis* L. and *Tilia* spp., exhibit sedative and anxiolytic effects, while *Crataegus monogyna* Jacq., *Leonurus cardiaca* L., and *Matricaria chamomilla* L. contribute through calming, cardiotonic, and adaptogenic actions. The mechanisms of action include, mainly, interactions with neurotransmitter systems (GABA, serotonin), antioxidant and anti-inflammatory effects, and the regulation of the stress response through the hypothalamic–pituitary–adrenocortical axis [13,14].

A relevant aspect is the use of these plants in phytotherapeutic combinations, in which the individual effects can mutually potentiate through synergistic mechanisms. Such phytocomplexes are considered advantageous due to their multitarget action and relatively favorable safety profiles compared to synthetic drug therapies. Regarding the origin of plant raw material, Romania has significant phytogeographic potential, offering optimal conditions for harvesting these species from spontaneous flora or from controlled cultures. However, in the context of pharmaceutical research and development, numerous plant-based products are patented at the international level, with patents mainly targeting extraction methods, standardization of active compounds, and pharmaceutical formulations. It is important to emphasize that patenting does not refer to the plant as a biological entity but to technological processes and finished products. Therefore, medicinal plants harvested in Romania can represent a valuable source of raw material, provided that the criteria of quality, botanical authentication, and absence of contaminants are met. However, for clinically validated therapeutic use, it is essential to integrate them into standardized products, obtained according to pharmaceutical norms and supported by scientific studies. In conclusion, the convergence between common pedoclimatic conditions, phytochemical composition, and scientific validation supports the use of these plant species as phytotherapeutic agents with anxiolytic and antidepressant effects, both in the traditional context and in that of modern medicine.

The novelty of this study lies in the integration of regional ethnobotanical data with modern pharmacological evidence and in the comparative analysis of selected species in the context of Romanian biodiversity, geographical distribution, and phytotherapeutic relevance. This approach allows for a more nuanced assessment of therapeutic potential and contributes to a better contextualization of the role of phytotherapy in contemporary strategies for the management of anxiety and depression disorders.

## 2. Materials and Methods

The present study constitutes a narrative review conducted using a classical, non-systematic approach to the analysis of the scientific literature concerning the use of medicinal plants from the Romanian wild flora in the management of anxiety and depression. The bibliographic search was performed using PubMed, Google Scholar, and Google Patents databases.

The search strategy included combinations of the following terms: “depression,” “anxiety,” “medicinal plants,” “phytochemical composition,” “adverse reactions,” “toxicity,” “mechanism of action,” “*Matricaria chamomilla* L.,” “*Galium odoratum* (L.) Scop.,” “*Melissa officinalis* L.,” “*Leonurus cardiaca* L.,” “*Hypericum perforatum* L.,” “*Tilia platyphyllos* Scop.,” “*Tilia tomentosa* Moench,” “*Tilia cordata* Mill.,” and “*Crataegus monogyna* Jacq.”

Both review articles and original research papers available in full-text format were included, the majority of which were published within the past 15–20 years. However, selected earlier studies considered relevant to the topic were also analyzed. The review encompassed both preclinical and clinical studies, with particular emphasis on efficacy, mechanisms of action, and safety profile.

## 3. Thematic Analysis of the Literature

### 3.1. Neurobiological and Clinical Foundations

Depression and anxiety disorders are among the most prevalent psychiatric conditions worldwide, characterized by a multifactorial etiology involving genetic, neurobiological, inflammatory, and psychosocial factors. From a clinical perspective, depressive disorder is defined as a complex affective condition marked by persistent depressed mood and/or loss of interest or pleasure (anhedonia), frequently accompanied by fatigue, sleep disturbances, appetite changes, impaired concentration, suicidal ideation, and feelings of worthlessness.

According to the Diagnostic and Statistical Manual of Mental Disorders, Fifth Edition (DSM-5-TR), these symptoms must persist for at least two weeks and result in clinically significant distress or impairment in personal, social, or occupational functioning in order for the diagnosis to be established [15]. Epidemiological studies estimate the lifetime prevalence of depressive disorders to range between 13% and 20%, with a higher incidence reported in women compared to men [5].

For several decades, the monoaminergic hypothesis has represented the dominant explanatory model of depression, proposing that deficiencies in serotonin, dopamine, and norepinephrine underlie affective symptomatology. Accordingly, serotonergic deficits have been correlated with depressed mood and irritability, dopaminergic hypofunction with anhedonia and reduced motivation, and noradrenergic dysfunction with fatigue and impaired concentration [16]. This framework provided the theoretical foundation for the development of selective serotonin reuptake inhibitors and other classes of antidepressants.

However, contemporary research has increasingly highlighted the limitations of this model. The delayed therapeutic onset of antidepressants, the substantial proportion of non-responders, and the lack of a direct correlation between plasma monoamine levels and clinical severity suggest that monoaminergic dysfunction alone is insufficient to account for the complexity of depressive disorders. Consequently, the monoamine hypothesis is currently regarded as partially valid but inadequate as a standalone explanatory model [17].

In recent years, research has focused on the imbalance between excitatory neurotransmission, mainly mediated by glutamate, and inhibitory neurotransmission, mediated by γ-aminobutyric acid (GABA), as a central mechanism in the pathogenesis of anxiety and depressive disorders. This imbalance affects the functioning of neural circuits involved in emotional and cognitive regulation, contributing to the appearance of affective symptoms [18]. However, it remains debatable whether these changes represent primary etiological factors or secondary consequences of chronic stress and hypothalamic–pituitary–adrenocortical (HPA) axis dysfunction.

The glutamatergic system is the main excitatory neurotransmitter system in the brain and is essential for cognitive processing. In depressed patients, neurochemical assessments have identified elevated basal glutamate levels in serum or plasma [19]. Glutamatergic hyperactivity, particularly through N-methyl-D-aspartate receptors (NMDARs), has been associated with neuronal excitotoxicity and synaptic dysfunction implicated in depression. In this context, various pharmacological agents, such as ketamine, have demonstrated rapid antidepressant effects. However, the clinical use of ketamine is limited by adverse effects and potential for abuse, highlighting the need to identify safer therapeutic alternatives [20].

Ketamine’s antidepressant effects are thought to be mediated by direct and indirect NMDAR inhibition, disinhibition of gamma-aminobutyric acid (GABA)-mediated interneurons, and conversion to the metabolite hydroxynorketamine (HNK). Together, these processes increase presynaptic glutamate release, with the net effect of increasing glutamatergic flux at the α-amino-3-hydroxy-5-methyl-4-isoxazole-propionic acid receptor (AMPAR) relative to the NMDAR [21].

On the other hand, the GABAergic system represents the main inhibitory mechanism of neuronal activity and is essential in maintaining the excitatory–inhibitory balance in the brain. Approximately one third of the synapses in the central nervous system involve GABAergic interneurons, which constitute 20–30% of neocortical neurons. These neurons express two major classes of receptors: GABA_A and GABA_B. GABA_A receptors are ionotropic, functioning as ligand-gated chloride channels and are predominantly located postsynaptically, where they mediate rapid synaptic inhibition. They are major pharmacological targets for numerous psychotropic agents, including benzodiazepines, barbiturates, and ethanol [22].

Structurally, GABA_A receptors are organized as pentameric complexes composed of various combinations of subunits (e.g., α, β and γ), arranged around a central pore permeable to chloride ions. In contrast, GABA_B receptors are metabotropic, coupled to Gi/o proteins, and are formed by a heterodimer consisting of the GABA_B1 and GABA_B2 subunits. They are located mainly at the presynaptic level, where they act as autoreceptors and modulate GABA release, but can also be found postsynaptically, contributing to the regulation of neuronal excitability [23].

GABAergic neurons contribute to the termination of the stress response by inhibiting the activity of the hypothalamic–pituitary–adrenocortical (HPA) axis. Dysfunction of this regulatory mechanism is associated with the negative effects of chronic stress exposure. For example, chronic stress causes a decrease in the expression of the K-Cl cotransporter (KCC2), which reduces the efficiency of inhibitory GABAergic transmission on neurons involved in the activation of the HPA axis [24].

In addition, genetic alterations of GABA_A receptors support the direct involvement of this system in affective disorders. Heterozygous deletion of the γ2 subunit causes decreased GABA_A receptor functionality and leads to hyperactivation of the HPA axis, being associated with anxiety-like and depressive-like behaviors [25]. Similar results have been reported in α2 subunit knockout mice [26].

Thus, these genetic models highlight the critical role of GABA_A receptors in emotional regulation and represent useful tools for investigating the mechanisms involved in anxiety and depression, as well as for developing new therapeutic strategies.

Hyperactivity of the HPA axis, characterized by chronically elevated cortisol secretion, represents another frequently reported central mechanism in both depression and anxiety. Prolonged exposure to elevated cortisol levels has been associated with hippocampal volume reduction and impaired prefrontal cortex function [27]. However, clinical studies indicate considerable interindividual variability in HPA axis dysregulation among patients.

In this context, the Dexamethasone Suppression Test (DST) was proposed as one of the earliest biological biomarkers in psychiatry, with initial studies reporting high specificity for melancholic depression. The initial enthusiasm reflected the hope of identifying an objective endocrine marker for diagnostic purposes. However, subsequent replication studies yielded inconsistent results and revealed considerable interindividual variability, limiting its clinical applicability and raising questions regarding the universality of hypothalamic–pituitary–adrenal (HPA) axis dysfunction in depression [28]. More recent research suggests that such variability may reflect the existence of distinct biological subtypes. For instance, studies employing the Trier Social Stress Test (TSST) have demonstrated divergent cortisol reactivity patterns depending on symptom profiles. In adolescents with major depressive disorder, reduced anticipatory cortisol reactivity has been observed compared to low-risk groups, and symptom dimensions appear to be differentially associated with endocrine response dynamics; affective symptoms correlate with heightened reactivity and rapid recovery, whereas neurovegetative symptoms are associated with hyporeactivity and delayed recovery [29]. These findings highlight a conceptual tension within the literature: while some studies support HPA axis hyperactivity as a central mechanism, others indicate hyporeactivity or subtype-dependent patterns. Thus, HPA axis dysfunction appears relevant for specific depressive phenotypes but does not constitute a universal biomarker. This biological variability limits the utility of endocrine markers as standalone diagnostic tools and supports the need for a dimensional and biologically informed framework in conceptualizing depression.

A growing body of evidence also implicates low-grade systemic inflammation in the pathogenesis of affective disorders. Elevated levels of proinflammatory cytokines, such as interleukin-6 (IL-6) and tumor necrosis factor-alpha (TNF-α), may influence tryptophan metabolism and reduce serotonin synthesis, promoting the accumulation of potentially neurotoxic metabolites along the kynurenine pathway. Concurrently, decreased levels of brain-derived neurotrophic factor (BDNF) have been associated with impaired neuroplasticity and increased vulnerability to stress [30]. Nevertheless, the causal relationship between inflammation and depression remains controversial, as it is unclear whether inflammation represents a primary etiological driver or a secondary consequence of chronic stress and associated behavioral alterations.

Closely related to depression, both clinically and neurobiochemically, anxiety disorders constitute another major group of psychiatric conditions with high population-level prevalence. Anxiety is characterized by a state of psychological and physiological hyperarousal, associated with persistent anticipation of negative events and heightened vigilance. From this perspective, anxiety may be conceptualized as a disorder of excessive stress-response regulation. Anxiety disorders—including generalized anxiety disorder, panic disorder, social anxiety disorder, and specific phobias—are among the most common psychiatric diagnoses worldwide [31].

From a neurobiochemical standpoint, anxiety is associated with dysfunction within neural circuits involved in fear and threat processing. Central to this is amygdala hyperactivity, a key structure in detecting and responding to threatening stimuli, accompanied by altered hippocampal and prefrontal cortex function—regions implicated in contextual regulation and top–down inhibition of emotional responses [32]. Imbalance among these structures contributes to the persistence of exaggerated anxiety reactions and impaired emotional control.

At the neurochemical level, anxiety has been linked to reduced GABAergic neurotransmission, resulting in diminished inhibitory tone and increased neuronal excitability. In parallel, serotonergic and noradrenergic systems play significant roles in the expression of anxiety symptoms, being indirectly involved in both vegetative manifestations—such as palpitations and sweating—and cognitive components, including excessive worry and catastrophic anticipation [33,34].

Similar to depression, anxiety disorders are frequently associated with hyperactivity of the hypothalamic–pituitary–adrenal (HPA) axis. Persistently elevated cortisol levels contribute to sustained tension, agitation, and hyperarousal, thereby facilitating symptom chronicity [35].

Clinically, anxiety disorders encompass a broad spectrum of manifestations, which may be grouped into somatic symptoms (palpitations, tremor, dyspnea), cognitive symptoms (intrusive thoughts, excessive worry, impaired concentration), and behavioral manifestations, such as avoidance of perceived threatening situations or dependency behaviors toward supportive individuals [36].

A central behavioral mechanism in anxiety disorders—particularly in specific phobias—is persistent avoidance of stimuli perceived as threatening. In these conditions, fear is linked to a specific object or situation, and symptom maintenance is commonly explained through classical conditioning processes, whereby a negative experience becomes associated with a particular stimulus. This association is subsequently reinforced by avoidance behaviors, which temporarily reduce anxiety but prevent fear extinction [37].

From a neurobiological perspective, phobias are correlated with amygdala hyperactivity and diminished prefrontal regulatory control over emotional responses, thereby facilitating disproportionate anxiety reactions [8,32,38]. A relevant example of a specific phobia is odontophobia (fear of dental treatment), a common condition with an estimated prevalence of 10–20% in the general population. Odontophobia often originates from prior experiences perceived as traumatic and the anticipation of pain in individuals with heightened anxiety vulnerability. The clinical consequences are significant, as avoidance of dental care leads to worsening oral pathology, amplification of anxiety, and progressive deterioration of oral health, with potential systemic repercussions [39].

Overall, contemporary research converges on the view that neither depression nor anxiety can be explained by a single neurobiological mechanism. Rather, these disorders reflect complex interactions among neuroendocrine, inflammatory, and synaptic systems, whose clinical expression varies across individuals. The absence of a robust universal biomarker does not represent a failure of biological research but rather underscores the necessity of a multimodal and biologically stratified approach. In this context, therapeutic interventions with multi-target mechanisms—including those derived from phytochemical sources—may be of theoretical interest; however, their validation requires rigorous standardization and systematic clinical confirmation.

The brain is affected by degenerative changes with age; thus, the biological aging phase is around the age of 65–70. Even in the case of the normal aging process, the activity of neurotransmitters (dopamine, serotonin, acetylcholine, noradrenaline, GABA, glutamate, etc.) is reduced; these are chemicals that ensure the transmission of nerve impulses between neurons. The etiopathogenic mechanism of phytocompounds (active plant compounds) in the treatment of depression and anxiety aims to regulate the neurochemical, hormonal, and inflammatory imbalances involved in these disorders. Phytocompounds act similarly to allopathic drugs, but often through multiple mechanisms and with fewer side effects. The etiopathogenesis of anxiety and depressive disorders is multifactorial, involving complex interactions between genetic, neurobiological, psychological, and environmental factors [40]. In generalized anxiety disorder, genetic vulnerability is supported by familial aggregation and increased concordance in monozygotic twins. Neurobiologically, hyperactivity of alert systems, including the amygdala and frontal cortex, is evident, along with dysfunction of neurotransmitters such as GABA and serotonin. Psychologically, intolerance of uncertainty and negative reinforcement mechanisms maintain symptomatology, and environmental factors modulate clinical expression [41].

Panic disorder has a significant genetic component and is associated with hypersensitivity to internal stimuli (e.g., CO_2_) and dysfunction of the serotonergic, GABAergic, and noradrenergic systems. The cognitive–behavioral model explains panic attacks by the catastrophic interpretation of somatic sensations, generating a vicious circle of anxiety. Psychiatric and somatic comorbidities frequently occur. Specific phobias develop through direct conditioning, observational learning, or informational transmission, while obsessive–compulsive disorder involves genetic, immunological, and neurochemical factors, with a central role of serotonin and the involvement of dopamine in repetitive behaviors. Hypochondriasis is explained by psychodynamic, cognitive, and learning models. In depression, the monoaminergic hypothesis highlights imbalances of noradrenaline, serotonin, and dopamine, complemented by GABAergic dysfunctions, alterations in the hypothalamic–pituitary–adrenal axis, and decreased neurotrophic factors (BDNF). Neuroimaging reveals cerebral hypometabolism, especially in the dorsolateral prefrontal cortex, and right temporal hyperactivity. Structurally, ventriculomegaly and subcortical changes are observed [42]. These disorders reflect integrated disorders of emotional and cognitive circuits, determined by the convergence of biological and psychosocial factors. In this context, the main etiopathogenic mechanisms addressed by phytocompounds consist of the following:

- Regulation of neurotransmitter systems: for example, phytocompounds in St. John’s wort modulate the level of neurotransmitters responsible for mood, such as serotonin (5-HT), noradrenaline (NA), and dopamine, by inhibiting their reuptake or inhibiting monoamine oxidase (MAO), thus increasing their availability in the synaptic cleft [43].
- Action on the GABAergic system: for example, quercetin can act on GABA (gamma-aminobutyric acid) receptors, the main inhibitory neurotransmitter in the brain, having a calming, sedative, and anxiety-reducing effect [44].
- HPA axis reduction: certain adaptogenic phytocompounds can help regulate cortisol levels (the stress hormone), reducing HPA axis hyperactivity, which is often dysfunctional in depression and chronic anxiety [45].
- Neuroprotective and anti-inflammatory effects: Bioactive compounds with antioxidant properties can reduce chronic inflammation and oxidative stress in the brain, factors that contribute to neurodegeneration and the appearance of depressive symptoms [46]—for example, chemazulene from chamomile [47]. Thus, phytocompounds act synergistically to restore neurochemical balance and reduce physiological stress, directly targeting the etiopathogenic mechanisms of anxiety and depression [48].

From this perspective, traditionally used medicinal plants may represent relevant examples of multimodal interventions, given the complexity of their phytochemical composition and their capacity to act simultaneously on multiple biological pathways. Recent research confirms that several traditionally employed species, such as St. John’s wort, hawthorn, and lemon balm, exhibit therapeutic properties supported by modern phytochemical and pharmacognostic investigations. The consolidation of these data provides not only a scientific framework for validating traditional uses but also a model for the systematic documentation of indigenous knowledge applicable to other regions.

### 3.2. Plants with Potential Anxiolytic and Antidepressant Effects

#### 3.2.1. Study Region

Romania is among the European Union countries characterized by high biogeographical diversity, resulting from the overlap of several distinct natural regions, including alpine, continental, Pannonian, Pontic, and steppe zones. This variety of geographical units, together with the characteristics of a temperate continental climate, creates favorable conditions for the development of a flora rich and diverse in composition. According to data reported in the scientific literature, the spontaneous flora of Romania comprises approximately 3829 vascular taxa and 979 non-vascular taxa, reflecting a high level of plant biodiversity [49,50].

Beyond this overall diversity, Romania’s flora is characterized by a significant presence of medicinal and aromatic plants traditionally used in folk medicine and currently integrated into modern phytotherapeutic practice. Phytobotanical assessments indicate the existence of over 750 plant species with medicinal properties within the country, underscoring the considerable potential of native vegetal resources for therapeutic applications and the development of phytopharmaceutical products [50].

This narrative review focuses on seven species frequently employed in Romanian traditional medicine for manifestations associated with anxiety and depression: chamomile (*Matricaria chamomilla* L.), sweet woodruff (*Galium odoratum* (L.) Scop.), lemon balm (*Melissa officinalis* L.), motherwort (*Leonurus cardiaca* L.), St. John’s wort (*Hypericum perforatum* L.), linden (*Tilia* spp.), and hawthorn (*Crataegus monogyna* Jacq.). Their selection was based on the frequency of traditional use, presence in the spontaneous flora of Romania, and availability of relevant phytochemical and pharmacological data supporting activity on the central nervous system. However, the level of scientific evidence supporting the anxiolytic and antidepressant effects varies considerably among species. While some, such as *Matricaria chamomilla* L., *Melissa officinalis* L., and *Hypericum perforatum* L., are supported by clinical studies [51,52,53], others, including *Leonurus cardiaca* L., *Hypericum perforatum* L., *Tilia tomentosa* Moench., and *Crataegus monogyna* Jacq., rely primarily on preclinical evidence [54,55,56], whereas *Galium odoratum* (L.) Scop [57] is mainly supported by traditional use.

Many of the plant species identified in the Romanian wild flora are also common throughout temperate regions of Europe [58]. In the context of the expanding “Green Medicine” trend, this study provides data that may support the development of phytotherapeutic products in accordance with European safety standards [59].

The geographical distribution of medicinal plant species is the result of a complex interaction between ecological, environmental, and historical factors, including climatic conditions (temperature and precipitation), altitude, soil type, and habitat, as well as biotic interactions and anthropogenic influences. In Romania, the diversity of ecosystems—from plains to mountainous and submontane regions—generates heterogeneous environmental conditions that shape the occurrence and abundance of species, as is well documented in biogeographical and species distribution modeling studies [60,61].

Romania is located in Southeastern Europe and has a temperate continental climate, characterized by relatively cold winters and warm summers. According to the Köppen–Geiger climate classification, the country is characterized by a humid continental climate with warm summers and a cold semi-arid climate [62]. Climate variability is influenced by the interaction of several regional atmospheric circulation centers (Mediterranean, Scandinavian-Baltic and Eastern European), which contributes to the variation in ecological conditions at the national level [63]. In this context, recent climate changes, characterized by an increase in the frequency of thermal extremes, a reduction in snow cover, and changes in precipitation patterns, may influence the distribution and dynamics of plant species, as well as the functioning of ecosystems [64].

**Figure 1:**
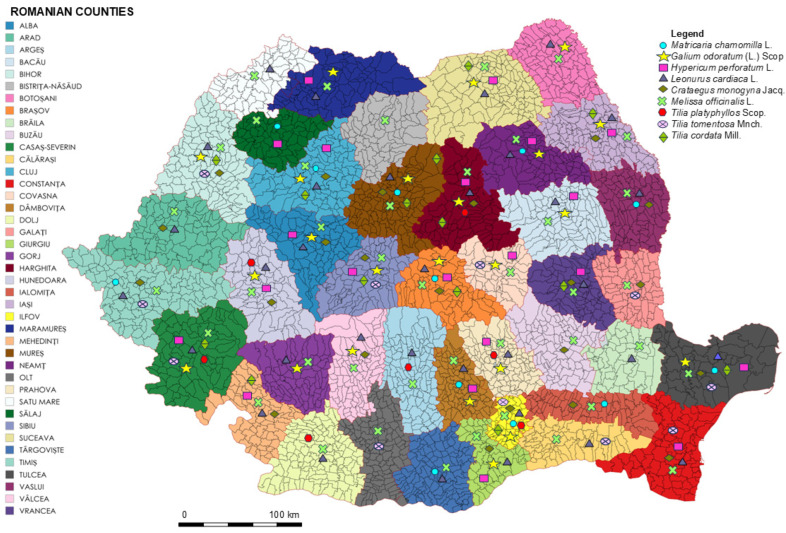
Geographical distribution of selected medicinal plant species in Romania (by counties) (figure created with MapChart version 5.9.0).

In this review, the species analyzed have a relatively wide distribution at the national level, being identified in multiple counties, especially in plains, hills, and submontane areas (Figure 1, Table S1 and Figure S1—Supplementary Material). However, the analysis of the distribution map highlights an uneven distribution, with a higher density of occurrences in the central regions of Romania, especially in the Transylvanian Colline Depression, as well as along the Subcarpathian chain. These regions are characterized by moderate climatic conditions (average annual temperature values ranging between 9 and 10 °C in the submontane areas and 6 and 9 °C in the hilly areas) and by favorable soils, such as Umbrisols, Spodosols, Cambisols, and Clay Visols. Plain regions are generally associated with low altitudes, higher temperatures (annual average values over 11 °C), and fertile soils, which is why they are subject to a higher degree of anthropization due to the intensification of agricultural activities and the transformation of natural habitats. This can lead to a reduction in the diversity and frequency of species in these areas. On the other hand, hilly and submontane areas represent transition regions that offer habitats conducive to the development of spontaneous flora, being characterized by moderate climatic conditions, which explains the higher frequency of records in these areas. In mountainous regions, temperatures are much lower (annual average values 0–4 °C), and the amount of precipitation is higher (annual average values 700–1200 mm), which can limit the development of a large number of species in these areas; this environment is specific to plants adapted to low temperatures, a short growing season, and increased abiotic stress [65].

The Carpathian Mountains play a key role in shaping species distribution, acting both as a geographical barrier and as a factor of ecological diversification, which can lead to population fragmentation and regional differences in their distribution. Also, the coexistence of several species in certain regions suggests the presence of favorable ecological niches and may reflect areas with a tradition of medicinal plant use.

These spatial variations are particularly relevant, because environmental factors influence the biosynthesis of secondary metabolites, potentially generating regional differences in the phytochemical composition and, implicitly, the biological activity and therapeutic efficacy of plants [66].

The medicinal plants *Matricaria chamomilla* L., *Galium odoratum* L., *Melissa officinalis* L., *Leonurus cardiaca* L., *Hypericum perforatum* L., *Tilia* spp., and *Crataegus monogyna* Jacq. fall within a common pedoclimatic area, specific to the temperate European zones, including the territory of Romania. These plant species develop in relatively similar soil conditions (chernozems and brown-acid soils), temperatures, and humidity regimes, factors that directly influence the biosynthesis of secondary metabolites and, implicitly, the phytochemical profile of the plants. The specialized literature highlights the fact that pedoclimatic parameters determine quantitative and qualitative variations in the bioactive compounds, such as flavonoids, phenolic acids, and volatile oils, responsible for the biological activity [13].

The distribution was estimated based on georeferenced occurrence data available in the Global Biodiversity Information Facility (GBIF) database. Only records containing valid geographic coordinates (hasCoordinate = TRUE), filtered for the territory of Romania, were selected. The data were processed using the WGS84 geodetic system and aggregated at the administrative level (county), to allow for the representation of the regional distribution of the analyzed species.

This approach allows for an objective estimate of distribution based on available observational data and is frequently used in biogeographic and ecological studies. Compared to other more complex geospatial or predictive modeling methods, it was preferred because GBIF is the largest data portal from which information on biodiversity, biogeographic (mapping), and taxonomic data can be collected from around the world, including Romania. The GBIF portal collects georeferenced data using custom web-scraping scripts in a batch process [67].

Cartographic representation was performed using an administrative map of Romania generated through the MapChart platform, with individual species differentiated by distinct graphical symbols. The resulting map was subsequently refined and supplemented with conventional cartographic elements using Microsoft PowerPoint 2024.

Knowledge of regional distribution allows for the identification of optimal areas for harvesting plants with high phytochemical potential, contributing to the standardization of raw materials and the development of effective phytotherapeutic products under sustainable exploitation conditions. Differences in distribution may also reflect intra-specific genetic variations, which may indirectly influence the biosynthesis of bioactive compounds.

The global distribution and extensive use of these species underline their therapeutic importance at the international level, while their inclusion in the Romanian flora provides a relevant framework for regional ethnopharmacological interpretation.

#### 3.2.2. Taxonomy and Botanical Description

The taxonomic characterization of the analyzed species holds not only descriptive but also pharmacognostic relevance, as affiliation with distinct botanical families (Asteraceae, Lamiaceae, Hypericaceae, Tiliaceae/Malvaceae, Rosaceae, Rubiaceae) correlates with different phytochemical profiles and, consequently, with distinct pharmacological mechanisms of action on the central nervous system. The literature indicates that both interspecific and intraspecific variability may significantly influence chemical composition.

A taxonomic classification of the plant species investigated as potential natural remedies for the management of anxiety and depression is presented in Table 1.

The plant species analyzed in the present study belong to distinct taxonomic groups within the angiosperms, characterized by considerable morphological and phytochemical diversity. Morphological diversity is reflected in variations in stem type, leaf structure, inflorescence organization, and fruit characteristics, aspects summarized in Table 2. These botanical features are essential for the accurate identification of plant raw materials and for preventing taxonomic misidentification or adulteration.

Beyond descriptive relevance, taxonomic and morphological differences can indirectly influence the chemical composition, quantity, and type of secondary metabolites, which can generate variations in the phytochemical profile and, implicitly, in the biological activity, which explains the lack of consistency of the results reported in the latter.

#### 3.2.3. Secondary Metabolites Identified in the Studied Plants

Medicinal plants have long been used in the management of depression and anxiety, with their therapeutic effects attributed to diverse pharmacognostic groups of bioactive compounds that act on multiple neurobiological targets. Species such as *Matricaria chamomilla* L., *Melissa officinalis* L., *Hypericum perforatum* L., *Leonurus cardiaca* L., *Tilia* spp., *Crataegus monogyna* Jacq., and *Galium odoratum* L. contain phytochemicals that modulate neurotransmission, neuroinflammation, and neuroendocrine responses.

Flavonoids represent one of the most important classes of active compounds in these plants. For example, apigenin from *Matricaria chamomilla* L. binds to benzodiazepine sites on GABA_A receptors, producing anxiolytic and mild sedative effects. Similarly, flavonoids from *Tilia* spp. and *Crataegus monogyna* contribute to central nervous system (CNS) depression and antioxidant activity. These compounds also reduce neuroinflammation and oxidative stress, which are increasingly recognized as contributing factors in mood disorders [81,82].

Phenolic acids, particularly rosmarinic acid in *Melissa officinalis* L., play a key role in anxiolytic activity. This compound inhibits GABA transaminase, leading to increased GABA levels in the brain. Additionally, *Melissa officinalis* L. extracts have been shown to modulate serotonergic pathways and enhance brain-derived neurotrophic factor (BDNF) expression, supporting neuroplasticity and resilience to stress [83]. Among the most extensively studied herbal antidepressants, *Hypericum perforatum* L. contains hyperforin and hypericin, which inhibit the reuptake of serotonin, dopamine, and noradrenaline. This mechanism resembles that of conventional antidepressants. Furthermore, *Hypericum* extracts modulate GABAergic and glutamatergic neurotransmission and may inhibit monoamine oxidase (MAO), thereby enhancing monoamine availability. Clinical evidence supports its efficacy in mild-to-moderate depression [81,84].

Alkaloids and iridoids present in *Leonurus cardiaca* L. contribute to its sedative and cardiotonic effects. Leonurine, a major alkaloid, is associated with modulation of autonomic function and reduction in somatic symptoms of anxiety, such as palpitations. Similarly, *Crataegus monogyna*, rich in oligomeric procyanidins and flavonoids, exerts cardioprotective and vasodilatory effects, indirectly alleviating anxiety by stabilizing cardiovascular function [81].

Coumarins, found in *Galium odoratum* L., exhibit mild CNS depressant activity and contribute to the plant’s traditional use as a sedative [70]. Essential oils present in *Matricaria chamomilla* L. and *Melissa officinalis* L. further enhance anxiolytic effects through the modulation of CNS excitability. Overall, these medicinal plants exert therapeutic effects through a combination of mechanisms, including enhancement of GABAergic transmission, inhibition of monoamine reuptake, reduction in oxidative stress, and modulation of the hypothalamic–pituitary–adrenal (HPA) axis. This multi-target activity supports their use as complementary treatments in anxiety and depressive disorders.

The chemical composition of the analyzed species, summarized in Table 3, is complex and predominantly characterized by biologically active secondary metabolites, including flavonoids, volatile oils, phenolic acids, sesquiterpene lactones, iridoids, and tannins. These compounds are recognized for their capacity to modulate central nervous system activity, contributing to the sedative, anxiolytic, and antidepressant effects attributed to these plant species.

Despite the promising results, the mechanisms of action of plant-derived compounds remain incompletely understood, and most evidence is derived from preclinical studies. Therefore, the proposed mechanisms should be interpreted with caution.

## 4. Selected Plant Species

**Figure 2:**
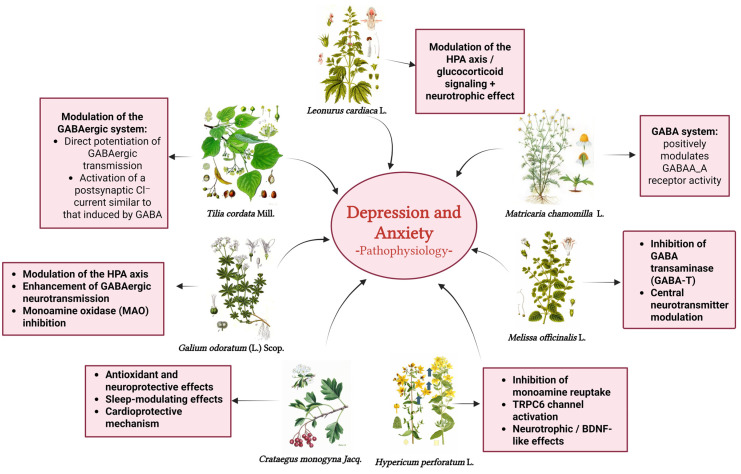
Mechanistic overview of medicinal plants in depression and anxiety. Created in BioRender. Daniela, F. (2026), https://BioRender.com/5x4mxg5 (accessed on 24 April 2026).

A schematic overview of the multitarget mechanisms of action of selected medicinal plants in the modulation of depression and anxiety is shown in Figure 2.

The figure illustrates the central role of depression and anxiety pathophysiology and highlights the main molecular pathways influenced by each species. *Hypericum perforatum* L. primarily acts through inhibition of monoamine reuptake and TRPC6 activation with neurotrophic effects; *Melissa officinalis* L. enhances GABAergic neurotransmission via GABA-transaminase inhibition; *Tilia cordata* Mill. and *Matricaria chamomilla* L. exert anxiolytic effects mainly through positive modulation of GABAA_A receptors; *Leonurus cardiaca* L. modulates the hypothalamic–pituitary–adrenal (HPA) axis and promotes neurotrophic pathways; *Galium odoratum* (L.) Scop. acts through a multimodal mechanism involving HPA axis regulation, GABAergic enhancement, and monoamine oxidase inhibition; and *Crataegus monogyna* Jacq. contributes through antioxidant, cardioprotective, and autonomic nervous system modulation, supporting stress reduction and improved sleep.

### 4.1. Matricaria chamomilla L.

#### 4.1.1. General Description and Distribution

*Matricaria chamomilla L*., a medicinal plant belonging to the Asteraceae family, is often referred to as the “star of medicinal plants” [166]. The species is also recognized under the scientific synonyms *Matricaria recutita* L. and *Chamomilla recutita* (L.) Rauschert, and represents one of the oldest and most widely used plants for therapeutic purposes. Commonly known as chamomile, it is also referred to as German chamomile, Roman chamomile, Hungarian chamomile, or English chamomile [167]. It is important to note that true chamomile is frequently confused with certain species of the genus *Anthemis*, particularly *Anthemis cotula* L., a toxic plant distinguished by its unpleasant odor [69]. *Matricaria chamomilla* L. is an annual herbaceous species, widely distributed in the spontaneous flora of Romania, traditionally used for its calming, anti-inflammatory, and anxiolytic properties [68].

#### 4.1.2. Level of Scientific Evidence

The mechanisms of action of *Matricaria chamomilla* L. are supported by preclinical and clinical data indicating anxiolytic effects and benefits in stress-related disorders [86].

#### 4.1.3. Phytochemical Composition

The plant contains, as can be seen in Table 3, a series of important bioactive compounds, such as flavonoids, phenolic acids, coumarins, azulenes, sesquiterpenes, and monoterpenes that contribute to its pharmacological activity [167].

#### 4.1.4. Mechanisms of Action

The precise mechanism underlying chamomile’s anxiolytic effects has not yet been fully elucidated. However, most studies suggest that among its flavonoid constituents, apigenin exerts sedative and anxiolytic effects through the modulation of γ-aminobutyric acid (GABA) receptors [168]. The plant also exhibits anti-inflammatory and antioxidant effects, helping to reduce oxidative stress involved in neuropsychiatric disorders.

#### 4.1.5. Preclinical Data

Experimental studies have demonstrated the anxiolytic, sedative, and anti-inflammatory effects of *Matricaria chamomilla* L. extracts, supporting the traditional use of the plant [86].

In a preclinical study, Ioniță et al. evaluated the anxiolytic and antidepressant effects of a hydroalcoholic chamomile extract in scopolamine-induced rat models using the elevated plus maze and forced swim tests. The findings suggested anxiolytic and antidepressant activity, potentially mediated through stimulation of the cholinergic system [169]. Moreover, a therapeutic formulation based on *Matricaria chamomilla* L. (CH12) was administered in bovines to reduce stress, inhibit cortisol production, and promote relaxation [170].

#### 4.1.6. Clinical Data

Clinical studies indicate benefits in reducing symptoms associated with stress and anxiety, as well as in improving some functional disorders. Thus, Amsterdam J.D. et al. conducted a randomized, double-blind, placebo-controlled trial at the University of Pennsylvania involving 179 subjects aged ≥ 18 years with a primary diagnosis of generalized anxiety disorder (GAD), with or without comorbid depression. Of these, 100 participants had GAD without depression, while 79 presented comorbid depressive symptoms. After eight weeks of treatment, depressive severity was assessed using the Hamilton Rating Scale for Depression (HRSD) and the Beck Depression Inventory (BDI). The results indicated that orally administered chamomile extract demonstrated significant anxiolytic effects and, in patients with GAD and comorbid depression, led to a significant reduction in depressive symptoms [171].

Additional evidence suggests that chamomile tea may alleviate anxiety symptoms in postmenopausal women [172] and may also reduce menstrual pain in women with dysmenorrhea [173].

#### 4.1.7. Safety, Toxicity, and Uses

*Matricaria chamomilla* L. is considered a safe species, frequently used in phytotherapeutic products [174].

According to studies, chamomile may interact with other herbs or various medications, resulting in adverse reactions. Theoretically, a combination of medications that affect platelet aggregation may interfere with the clotting effect, thus increasing the risk of bleeding. In addition, high doses of chamomile pose a teratogenic risk, affect the menstrual cycle, and cause vomiting. Although this herb has antiallergic activity, several studies have reported allergic reactions such as contact dermatitis and hypersensitivity, especially in people allergic to pollen or composites. There is a possibility of reducing follicular function and development, leading to premature birth in pregnant women when using chamomile [85]. Although no side effects were reported in several articles, and most symptoms did not require specialized medical intervention, some studies conducted on pregnant women have shown that consuming chamomile during pregnancy can lead to dangerous outcomes, both for the mother and the newborn. [168]. Some studies suggest that regular consumption during pregnancy may be associated with adverse perinatal outcomes, including small-for-gestational-age (SGA) neonates and reduced birth length [175]. Chamomile has been described as possessing oxytocic and uterine-stimulating properties, which may theoretically increase the risk of spontaneous abortion or preterm birth and has also been reported as a potential trigger of severe anaphylaxis, which can give rise to immediate type I reactions [176,177]. Allergenic proteins present in chamomile extracts may cross-react with other *Compositae* species [178,179]. Potential drug interactions have also been suggested as side effects of chamomile use; for example, coumarin derivatives in chamomile may interfere with the blood clotting process, with aspirin, anti-inflammatory drugs, and antidepressants. [168]. Regarding toxicity, very few studies have been conducted. Thus, Kalantari H. et al., using the micronucleus test in peripheral blood reticulocytes, observed that among the products tested, the chamomile-based one presented unclear results, suggesting a possible genotoxic risk but requiring further investigation. [174].

### 4.2. Galium odoratum (L.) Scop.

#### 4.2.1. General Description and Distribution

*Galium odoratum* (L.) Scop. is a perennial herbaceous species, found mainly in forest areas in Romania, traditionally used for its sedative and calming properties.

*Galium odoratum* (L.) Scop. belongs to the botanical genus Galium, commonly known in Romania as “drăgaică” or “sânziene,” which comprises over 38 species. Among the most recognized species are *Galium aparine* L. (cleavers)*, Galium verum* L. (lady’s bedstraw), *Galium odoratum* (L.) Scop. (sweet woodruff), *Galium mollugo* L., *Galium purpureum* DC., *Galium rubioides* L., and *Galium schultesii* Vest [71].

*Galium odoratum* (L.) Scop., formerly known as *Asperula odorata* (L.), is a perennial herbaceous plant native to Europe and Western Asia, widely distributed in moist, shaded forests across regions of Russia, Africa, China, Japan, Europe, and Romania [71,107].

The leaves and flowers give a sweet, hay-like scent, particularly pronounced when freshly cut. Coumarin is the principal compound responsible for this sweet, characteristic scent [70,71] and is formed during the drying process from melilotoside [71,72,73].

#### 4.2.2. Level of Scientific Evidence

Galium odoratum is supported primarily by phytochemical and preclinical studies, with limited evidence of clinical effects [73,107].

#### 4.2.3. Phytochemical Composition

The plant contains bioactive compounds such as coumarins, flavonoids, and phenolic acids (Table 3), which contribute to the biological activity [71].

#### 4.2.4. Mechanisms of Action

Sedative and anxiolytic effects are associated with the presence of coumarins and phenolic compounds, which can influence the central nervous system and the stress response [107].

The mechanisms of action of this medicinal plant, underlying the traditional use in affective disorders, appear to partially converge with those described for *Hypericum perforatum* L. However, unlike *H. perforatum* L., where monoamine reuptake inhibition represents a central mechanism, the effects of sweet woodruff on the central nervous system are predominantly attributed to its sedative, anti-inflammatory, and neuroprotective properties, suggesting a modulatory rather than direct synaptic antidepressant action.

Available experimental data indicate that iridoids, coumarins, and flavonoids identified in the plant influence neuroendocrine and inflammatory pathways. Iridoids and flavonoids have been shown to modulate the hypothalamic–pituitary–adrenal (HPA) axis, leading to reduced secretion of corticotropin-releasing hormone (CRH) and adrenocorticotropic hormone (ACTH) through attenuation of stress-induced cortisol elevation—an effect particularly relevant in the context of HPA axis dysregulation observed in depression. Concurrently, coumarins may exert anxiolytic and antidepressant-like effects via enhancement of GABAergic neurotransmission and inhibition of monoamine oxidase (MAO), contributing to emotional stabilization and reduced neuronal hyperexcitability [180].

#### 4.2.5. Preclinical Data

Experimental studies have revealed the antioxidant effects and anxiolytic potential of extracts of *Galium odoratum* (L.) Scop. [107]. Administration of a dried extract of *Galium odoratum* (L.) Scop. in mice significantly reduced locomotor activity in an open-field test, an effect interpreted as sedative and depressant in the central nervous system, without impairing motor coordination. This behavioral profile suggests central depressant activity with potential secondary anxiolytic effects, although direct receptor involvement has not been conclusively established [11]. Convergent findings have been reported in standardized anxiety models, including the elevated plus maze and light–dark box tests, where woodruff extracts increased time spent in non-aversive zones. However, these results should be interpreted cautiously due to potential confounding effects related to reduced locomotor activity [181].

Regarding antidepressant activity, more robust pathophysiological evidence derives from studies on asperuloside, an iridoid glycoside present in *Galium odoratum* (L.) Scop. In chronic unpredictable stress-induced depression models in rats, asperuloside reduced depressive-like behaviors and improved cognitive performance. These effects were associated with activation of the Wnt3α/GSK-3β/β-catenin signaling pathway, a molecular cascade implicated in neuroplasticity and stress adaptation. Experimental inhibition of β-catenin abolished the antidepressant-like effects of asperuloside, supporting a causal relationship between this pathway and the observed behavioral outcomes [182].

Additionally, recent research has demonstrated that asperuloside exerts central anti-inflammatory effects by inhibiting nuclear factor kappa B (NF-κB) activation through stabilization of its inhibitor IκBα via increased O-GlcNAcylation. This molecular modulation was associated with reduced neuronal apoptosis in the hippocampus, a brain region critically involved in depression pathophysiology [183]. Furthermore, Razzivina et al. reported that *Galium odoratum* (L.) Scop. extracts possess antioxidant and immunomodulatory properties, reflected in reduced macrophage polarization toward the pro-inflammatory M1 phenotype. These effects were correlated with phenolic compounds such as chlorogenic acid, flavonoids, and coumarins, which may indirectly contribute to anxiolytic and antidepressant effects by attenuating oxidative stress and systemic inflammation [72].

#### 4.2.6. Clinical Data

Clinical data are limited, and further studies are needed to confirm the therapeutic effects.

#### 4.2.7. Safety, Toxicity and Uses

*Galium odoratum* (L.) Scop. is traditionally used in phytotherapy, but data on safety and toxicity are limited, requiring caution in use.

In Bulgaria, *Galium odoratum* L. is included on the official list of herbaceous plants and flowers with toxic potential. Reported adverse effects include dizziness, fatigue, headache, vomiting, abdominal colic, convulsions, respiratory difficulty, and, in severe cases, paralysis. The toxicity of *Galium odoratum* (L.) Scop. is primarily attributed to its coumarin content [57]. Historically, coumarin has been classified as a potentially toxic compound. A report published by the U.S. Food and Drug Administration (FDA) in 1954 indicated that coumarin induced hepatic tumors in experimental rat models [184]. Based on rodent toxicological studies, the National Institute for Occupational Safety and Health (NIOSH) categorized coumarin among chemical agents with carcinogenic potential. Consequently, the FDA prohibits the intentional addition of coumarin to food products [185] and advises against its concomitant use with medications affecting circulation or during pregnancy.

Regulatory approaches vary between countries. Although *Galium odoratum* (L.) Scop. is listed as a plant with toxic effects in Bulgaria, in Germany it may be used in food products within controlled limits [57]. Direct experimental data regarding toxicity following human consumption of *Galium odoratum* (L.) Scop. remain scarce, and the presumed risk is largely extrapolated from known coumarin toxicity profiles. The German Federal Institute for Risk Assessment (BfR) established a tolerable daily intake (TDI) for coumarin of 0.1 mg/kg body weight/day [186]. The European Food Safety Authority (EFSA) further indicated that short-term intake up to three times the TDI is unlikely to pose a significant health risk, provided that exposure does not exceed 1–2 weeks [187]. Based on these data, it has been estimated that a 70 kg adult may safely consume approximately 7 g of fresh *Galium odoratum* (L.) Scop. flowers, assuming typical coumarin concentrations [57].

### 4.3. Melissa officinalis L.

#### 4.3.1. General Description and Distribution

*Melissa officinalis* L. is a perennial species, traditionally used for its calming and anxiolytic properties, being frequently found in the spontaneous and cultivated flora of Romania [68].

#### 4.3.2. Level of Scientific Evidence

*Melissa officinalis* L. is supported by preclinical and clinical data indicating anxiolytic, sedative effects and benefits on sleep and cognitive function [188,189].

#### 4.3.3. Phytochemical Composition

The phytochemical composition of the plant includes (Table 3) bioactive compounds such as phenolic acids, flavonoids, pentacyclic triterpenic acids, monoterpenes, and the sesquiterpenes responsible for the pharmacological activity [190].

#### 4.3.4. Mechanisms of Action

The anxiolytic and sedative effects of lemon balm are correlated with influencing the central nervous system and reducing oxidative stress [191]. The plant is also associated with improving cognitive function and reducing stress through mechanisms involving central neurotransmitters [189,190].

**Figure 3:**
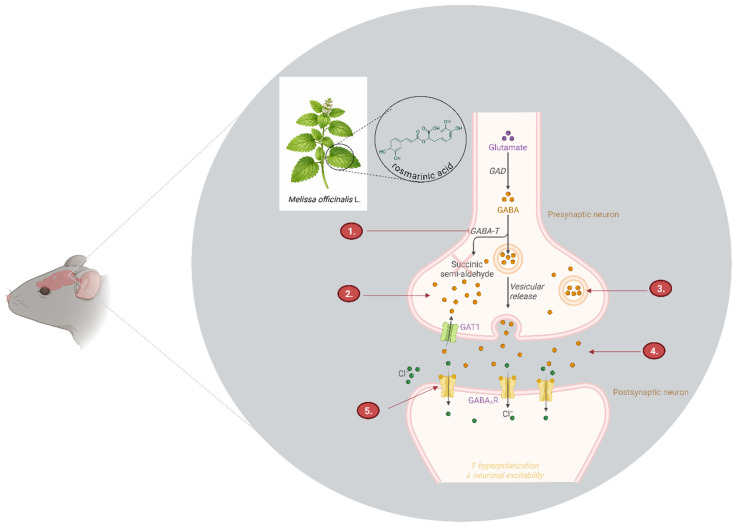
Possible (preclinical) mechanism of action of rosmarinic acid present in extracts of the *Melissa officinalis* L. plant in rat models. Created in BioRender. Daniela, F. (2026), https://BioRender.com/reds3rl (accessed on 20 April 2026).

Pharmacological studies have demonstrated that *Melissa officinalis* L. extracts inhibit γ-aminobutyric acid transaminase (GABA-T) activity (Figure 3), thereby reducing GABA degradation and increasing synaptic availability of this inhibitory neurotransmitter. This mechanism supports the hypothesis that lemon balm exerts anxiolytic and sedative effects through enhancement of GABAergic tone, functionally resembling the mechanism of action of certain synthetic anxiolytic agents [192]. Preclinical studies have specifically implicated rosmarinic acid in the modulation of the GABAergic system. Rosmarinic acid may inhibit GABA-T (1), leading to increased intracellular GABA concentrations at the presynaptic level (2). This may indirectly promote GABA accumulation within presynaptic vesicles (3) and enhance its availability for release into the synaptic cleft (4). Once released, GABA binds to GABA__A_R, receptors via passive diffusion, triggering chloride (Cl^−^) channel opening and neuronal hyperpolarization (5). This cascade ultimately contributes to reduced neuronal excitability and attenuation of anxiety-related symptoms.

A central role in these effects is attributed to rosmarinic acid, the principal phenolic compound of lemon balm, recognized for its antioxidant and anti-inflammatory activity. Rosmarinic acid may indirectly contribute to anxiolytic and antidepressant effects through reduction in neuroinflammation and mitigation of HPA axis dysregulation, a mechanism also relevant to the action of iridoids in *Galium odoratum* (L.) Scop. Although lemon balm has not been associated with direct activation of specific ion channels such as TRPC6 (as described for hyperforin), its anti-inflammatory and antioxidative properties suggest an indirect influence on neuroplasticity and neuronal resilience [193].

#### 4.3.5. Preclinical Data

With regard to antidepressant effects, evidence derives predominantly from preclinical studies. Animal models of depression have shown that hydroalcoholic extracts of *Melissa officinalis* L. reduced depressive-like behaviors in the forced swim test and tail suspension test. These effects were associated with modulation of monoaminergic systems—particularly serotonergic and noradrenergic pathways—as well as with attenuation of oxidative stress in brain tissue [191].

#### 4.3.6. Clinical Data

Clinical studies have reported beneficial effects of *Melissa officinalis* L. on anxiety symptoms associated with mild cognitive impairment and Alzheimer’s disease, suggesting a favorable impact on both cognitive and emotional domains. These effects have been attributed to additional mechanisms, including cholinergic interactions and antioxidant properties, further supporting the hypothesis of a multifaceted neuroprotective profile [194].

With regard to sleep disturbances, significant improvements were reported in a double-blind, placebo-controlled clinical trial involving 80 patients who received 3 g/day of *Melissa officinalis* L. capsules for eight weeks. In addition to improved sleep quality, reductions in anxiety and depressive symptoms were also observed [188]. Similarly, in menopausal women, administration of 500 mg/day of *Melissa officinalis* L. for eight weeks in a randomized, double-blind, placebo-controlled study resulted in significant improvement in sleep disturbances [195].

The anxiolytic efficacy of *Melissa officinalis* L. is supported by several controlled clinical studies. Kennedy D.O. et al. demonstrated that administration of *Melissa officinalis* L. extract to healthy volunteers reduced stress-induced anxiety and improved subjective calmness, alongside favorable changes in cognitive performance, suggesting a direct central effect [196]. Similar findings were reported in a randomized, double-blind, placebo-controlled trial in which lemon balm significantly reduced anxiety levels and psychological tension under experimentally induced acute stress conditions [189].

#### 4.3.7. Safety, Toxicity, and Uses

A clinical study reported adverse reactions, including vomiting, dizziness, respiratory wheezing, agitation, abdominal pain, and nausea following administration of *Melissa officinalis* L. extract at a dose of 60 drops/day. However, statistical analysis did not reveal significant differences between the treatment and placebo groups regarding the frequency of these effects. Other adverse reactions described in the literature include appetite stimulation, headache, alterations in electroencephalographic activity at high doses (1200 mg), reduced alertness at 900 mg, increased intraocular pressure, palpitations, and potential modulation of thyroid hormone secretion [75]. Topical application has been associated with local reactions such as erythema, contact dermatitis, burning sensation, paresthesia, residual hyperpigmentation, and skin irritation. In this context, caution is warranted when administering *Melissa officinalis* L. products at high doses or over prolonged periods, pending more robust chronic toxicity and dose–response data [75].

In vitro toxicological studies provide additional insight. Essential oil of *Melissa officinalis* L. was found to be safe for human epithelial type 2 (HEp-2) cells at concentrations below 100 mg/mL but became cytotoxic at higher concentrations, as evidenced by increased trypan blue uptake indicating cell death. Similarly, Mahita et al. demonstrated a dose-dependent effect of lemon balm essential oil on neuronal cells. Low concentrations (<0.001 mg/mL) exerted modest neuroprotective effects (≈10% increase in viability), whereas higher concentrations (>0.01 mg/mL) induced neurotoxicity (≈20% reduction in viability), potentially associated with GABA_A_ receptor inhibition [197]. In vivo studies have shown that after oral administration to rats, lemon balm essential oil did not produce toxicity, if used in doses of 300 and 2000 mg/kg [198], nor in Swiss albino rats at doses of 2000 mg/kg [199]. Extracts of *Melissa officinalis* L., including an ethanolic extract administered in doses of 250 and 500 mg/kg, and an aqueous extract administered at 100 mg/kg, in mice, for 2 weeks, did not produce genotoxic or mutagenic effects [200]. Clinical studies consider *Melissa officinalis* L. to be safe and well tolerated by the body, both in cases of oral and topical administration, presenting a minimal risk of side effects or serious adverse reactions, even in significant doses or in combination with other plants. Thus, Shakeri A. et al. claims that dried lemon balm powder standardized in rosmarinic acid was well tolerated by the body after oral administration for 8 weeks, being rapidly absorbed (CMax in the blood reached after approximately one hour) [75]. Rosmarinic acid has been detected in serum using coulometric detection coupled with high-performance liquid chromatography [201]. Nevertheless, some findings raise concerns regarding chronic toxicity. A study in rats demonstrated that oral administration of hydroalcoholic extract of *Melissa officinalis* L. at 600 mg/kg for 30 days induced significant hepatic and renal lesions, accompanied by unfavorable alterations in serum biochemical parameters [202]. Regarding essential oil toxicity, the reported LD_50_ in mice is approximately 2.57 g/kg. However, oral doses exceeding 1 g/kg or prolonged administration have been associated with adverse effects on organ structure and function, particularly in the liver and kidneys [203].

### 4.4. Leonurus cardiaca L.

#### 4.4.1. General Description and Distribution

*Leonurus cardiaca* L. (motherwort) is a perennial herbaceous species, widespread in the spontaneous flora of Romania, traditionally recognized for its sedative, hypotensive, and cardiotonic effects.

#### 4.4.2. Level of Scientific Evidence

*Leonurus cardiaca* L. is mainly supported by preclinical data and phytochemical studies, with limited clinical evidence of anxiolytic effects [125,126].

#### 4.4.3. Phytochemical Composition

The plant contains bioactive compounds such as flavonoids, phenolic acids, alkaloids (leonurines), sesquiterpenes, monoterpenes, and pentacyclic triterpenic acids (Table 3) that contribute to its pharmacological activity [126].

#### 4.4.4. Mechanisms of Action

The effects of *Leonurus cardiaca* L. are associated with actions on the central nervous system, including sedative and anxiolytic effects, as well as modulation of the cardiovascular system [204]. Bioactive compounds can also influence the stress response and neurovegetative balance.

**Figure 4:**
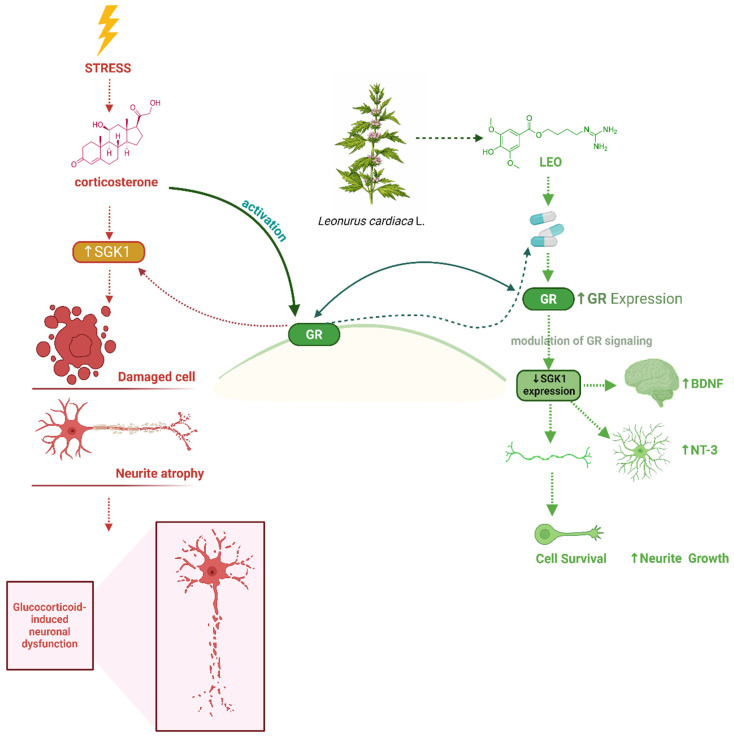
Proposed in vitro mechanism of leonurine (LEO) in corticosterone-exposed neuronal cells. Created in BioRender. Daniela, F. (2026), https://BioRender.com/uy7b56o (accessed on 20 April 2026). Legend: SGK1—serum and glucocorticoid regulated kinase 1; GR—glucocorticoid receptor; LEO—leonurine; BDNF—Brain-Derived Neurotrophic Factor; NT-3—Neurotrophin-3.

Leonurine is widely regarded as the principal bioactive compound of *Leonurus cardiaca* L. and is considered largely responsible for its anxiolytic and antidepressant-like effects. In vitro studies using PC12 neuronal cells exposed to corticosterone to induce a depression-like state demonstrated that leonurine exerted a concentration-dependent protective effect (10, 20, 40, 60, 80, and 100 μM, for 24 h, with maximal pro-survival activity observed at 60 μM). As can be seen in Figure 4, exposure to corticosterone was associated with increased SGK1 expression, accompanied by neuronal morphological alterations and neurite atrophy. Treatment with leonurine (LEO) appeared to increase glucocorticoid receptor (GR) expression while attenuating SGK1 levels. This regulatory effect was further associated with the upregulation of neurotrophic factors, including BDNF and NT-3, which may contribute to improved neuronal viability and enhanced neurite outgrowth. Overall, these findings suggest that LEO may modulate glucocorticoid-related signaling pathways under in vitro conditions [205].

#### 4.4.5. Preclinical Data

Pharmacological investigations suggest that the anxiolytic effects of *Leonurus cardiaca* L. may be mediated through modulation of the GABAergic system. Extracts of the plant have been shown to inhibit the binding of the radioligand [(^3^)H]-SR95 531, which specifically targets the GABA binding site of the GABA_A receptor. This inhibitory effect increased in a concentration-dependent manner and was attributed primarily to leonurine, which demonstrated an IC_50_ of 15 μg/mL, compared to an IC_50_ of 21 μg/mL for the crude extract [126].

Further receptor-binding studies conducted in rats using *Leonurus cardiaca* L. and *Leonurus japonicus* L. extracts demonstrated direct action at the GABA binding site, with significantly weaker interaction at the benzodiazepine site, suggesting selective modulation of the GABAergic system [206].

#### 4.4.6. Clinical Data

Clinical data are limited, but traditional use and presence in phytotherapeutic products suggest therapeutic potential in anxiety and cardiovascular disorders.

In a clinical study involving 50 patients with stage 1 and 2 arterial hypertension accompanied by anxiety and sleep disturbances, administration of 1200 mg/day of *Leonurus cardiaca* L. essential oil for 28 days resulted in improvement of psycho-emotional status and reduction in blood pressure values. According to the Clinical Global Impression (CGI) scale, anxiety and depressive symptoms improved markedly in 32% of patients, moderately in 48%, and slightly in 8%, while 12% showed no therapeutic response [207].

#### 4.4.7. Safety, Toxicity, and Uses

*Leonurus cardiaca* L. is frequently used in food supplements and phytotherapeutic products with sedative and anxiolytic effects, having a favorable safety profile under correct use conditions.

The use of *Leonurus cardiaca* L. preparations is generally well tolerated at therapeutic doses; however, the literature reports several clinically relevant adverse effects. One of the most important concerns is an increased risk of bleeding, attributed to the plant’s antiplatelet activity. *Leonurus cardiaca* L. has been associated with “bleeding abnormalities,” a particularly important consideration in the perioperative setting [208]. Reviews addressing herb–drug interactions further indicate the potential for potentiation of anticoagulant therapy, especially warfarin, thereby increasing the risk of hemorrhagic complications [126,209].

At the gastrointestinal level, mild digestive disturbances such as diarrhea and gastric irritation have been reported, particularly with higher doses or concentrated extracts. Although *Leonurus cardiaca* L. is not classified as a strong sedative, excessive sedation has been described when administered concomitantly with benzodiazepines or other central nervous system depressants, likely due to synergistic effects [210,211]. Cutaneous adverse reactions, including contact dermatitis and photosensitization, have also been reported, especially following direct contact with fresh leaves or crude extracts [212].

Wellington E.O. et al. reported that oral administration of aqueous extract of *Leonurus cardiaca* L., at doses up to 500 mg/kg, for a period of 21 days, to albino Wistar rats, was not associated with mortality or with the appearance of histopathological lesions in vital organs. Moreover, histological examinations revealed the maintenance of structural integrity, as well as a possible improvement in tissue architecture in the liver, kidney, and heart. However, statistically significant changes were recorded in some hematological parameters (RBC, Hb, MCV, WBC), plasma electrolyte balance, and liver enzyme activity (ALT, AST, ALP). These variations indicate systemic biological effects induced by the extract, without suggesting, however, clear manifestations of toxicity under the experimental conditions used [213].

### 4.5. Hypericum perforatum L.

#### 4.5.1. General Description and Distribution

*Hypericum perforatum* L. (St. John’s wort) is a perennial, glabrous, erect plant, typically woody at the base. Its leaves contain numerous translucent secretory glands that appear as perforations when viewed against light, a characteristic that explains the species epithet “perforatum” [68,78,79].

*Hypericum perforatum* L. is one of the most studied medicinal plants, being widespread in the spontaneous flora of Romania and traditionally used for its antidepressant and anxiolytic properties [148].

#### 4.5.2. Level of Scientific Evidence

*Hypericum perforatum* L. is supported by numerous preclinical and clinical studies, being one of the best documented species used in the treatment of depression [214,215].

#### 4.5.3. Phytochemical Composition

The plant contains important bioactive compounds from various classes—naphthodianthrones (hypericin and pseudohypericin), phlorogluninols (hyperforin), flavonoids, phenolic acids, and other compounds responsible for the pharmacological effects [134,135]. Although numerous clinical studies have attributed the antidepressant efficacy of *H. perforatum* L. primarily to hyperforin content [216], other constituents—including hypericin, pseudohypericin, flavonoids, and oligomeric procyanidins—may also contribute directly or indirectly to the pharmacological activity overall [214].

#### 4.5.4. Mechanisms of Action

The antidepressant effects of *Hypericum perforatum* L. are mainly associated with the inhibition of monoamine reuptake (serotonin, dopamine, and noradrenaline), a mechanism that contributes to improving mood [214].

The plant also influences neurotransmitter systems and neuroplasticity processes involved in the pathogenesis of depression [215]. According to Nathan P.J., the mechanism of action of *Hypericum perforatum* L. in depression and anxiety involves inhibition of monoamine reuptake, including serotonin, dopamine, and norepinephrine, as well as modulation of amino acid neurotransmitters such as GABA and glutamate. Unlike classical monoamine reuptake inhibitors, St. John’s wort appears to exert this effect in a non-competitive manner, a mechanism attributed to increased intracellular sodium (Na^+^) concentrations. At the receptor level, prolonged administration of *Hypericum* preparations has been associated with downregulation of β_1_-adrenergic receptors and upregulation of postsynaptic 5-HT_1_A and 5-HT_2_ receptors [214].

Recent evidence from Otero M.C. highlights that the molecular mechanisms underlying hyperforin’s antidepressant effects are not yet fully elucidated. Some studies suggest that hyperforin activates transient receptor potential canonical 6 (TRPC6) channels, which are expressed in the hippocampus and other cortical regions [215]. Experimental TRPC6 deficiency in mice according to Hamdaoui E.Y. et al., has been shown to induce anxiety- and depression-like behaviors, accompanied by reduced excitability of CA1 hippocampal pyramidal neurons [217]. These findings support the hypothesis that hyperforin-mediated TRPC6 activation may partially replicate brain-derived neurotrophic factor (BDNF)-like effects, given that BDNF regulates both the function and expression of TRPC6 channels.

Moreover, a specific binding region for hyperforin has been identified within the C-terminal segment of TRPC6; mutations at this site abolish channel responsiveness to hyperforin. Notably, hyperforin does not activate closely related TRPC family members, such as TRPC3 or TRPC7, indicating a relatively high degree of selectivity for TRPC6 [215,217,218].

**Figure 5:**
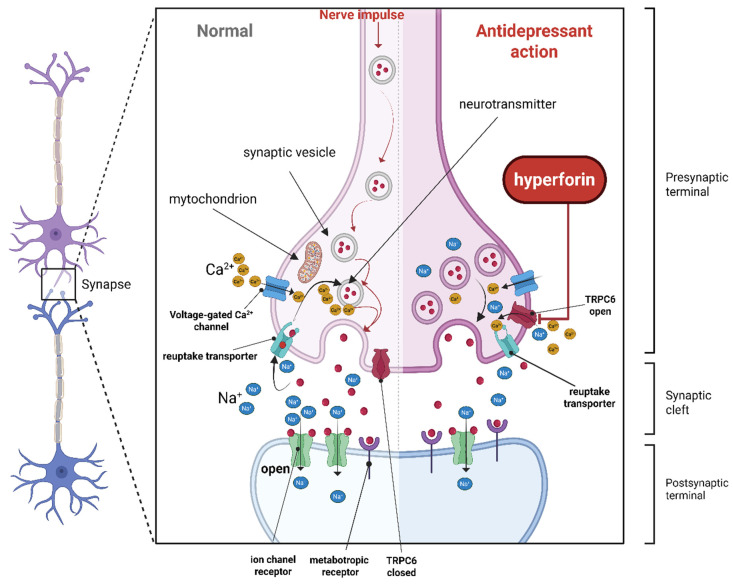
Mechanism of action of hyperforin in depression, performed on type Human Embryonic Kidney 293 (HEK293) and rat pheochromocytoma (PC12) cell lines. Created in BioRender. Daniela, F. (2026), https://BioRender.com/pzl5gd7 (accessed on 20 April 2026). Legend: TRPC6—Canonical Transient Receptor Potential 6 Channel.

A proposed mechanistic model (Figure 5) suggests that hyperforin may indirectly inhibit monoamine reuptake through TRPC6 activation. Opening of these ion channels increases intracellular Na^+^ concentration, thereby reducing the sodium gradient across the synaptic membrane. As monoamine transporters rely on this gradient for neurotransmitter reuptake, diminished gradient strength impairs their ability to transport monoamines from the synaptic cleft back into the presynaptic neuron, leading to increased synaptic availability [219,220].

Clinical evidence supporting efficacy is relatively robust; however, the risk of drug–herb interactions is significant and well documented. Clinical recommendations should therefore include explicit warnings regarding cytochrome P450 (CYP) enzyme induction, particularly CYP3A4, as well as potential effects on oral contraceptives and other critical medications, including immunosuppressants, anticoagulants, antiretrovirals, and certain psychotropic agents. Longitudinal studies and comprehensive pharmacovigilance assessments are warranted to further delineate the long-term safety profile and interaction spectrum of *Hypericum perforatum* L. in routine clinical use.

#### 4.5.5. Preclinical Data

In an experimental study conducted on mice, the effects of *Hypericum perforatum* L. extract were investigated in a model of anxiety and depression induced by chronic corticosterone administration over a period of 7 weeks. The results of the study showed that chronic corticosterone treatment induced anxious/depressive behavior, associated with decreased proliferation of progenitor cells and reduced dendritic spine density. Administration of *Hypericum perforatum* L. at doses of 30 mg/kg for 3 weeks reversed these effects, normalizing behavior and preventing the decrease in hippocampal neurogenesis, as well as the loss of synaptic connectivity.

These effects suggest that the mechanism of action of St. John’s wort is not only neurochemical but also neurotrophic and neuroplastic, involving the stimulation of neurogenesis in the hippocampus and the restoration of dendritic structure. Through these morphological adaptations, *Hypericum perforatum* L. contributes to increasing neuronal resilience to chronic stress, explaining its antidepressant and anxiolytic effects observed experimentally [221].

#### 4.5.6. Clinical Data

In a randomized clinical trial of *Hypericum perforatum* L. with 80 postmenopausal women (45–60 years old) equally divided into two groups—a treatment group and a placebo group—only 70 of them completed the 8-week intervention. The participants received between 270 and 330 μg of St. John’s wort extract three times a day. The patients’ condition was assessed using the modified Kupperman index for menopausal symptoms (including hot flashes), applied at regular intervals throughout the study, and the severity of depression was measured using the Hamilton Depression Rating Scale at baseline and at the end. The results showed a significant reduction in the frequency and intensity of hot flashes, the menopause score, and depression in the treatment group; 80% of the participants were free of depression at the end of treatment, compared to 5.7% in the placebo group [222].

A prospective observational study evaluated the efficacy of a tincture prepared from the fresh plant of *Hypericum perforatum* L. in patients with mild-to-moderate depression, compared with patients included in randomized controlled trials with high-dose dried extracts. The study included 52 patients, of whom 51 were evaluated (1 dropout) and 42 who completed the full 6-week evaluation. The results showed a reduction in the HAM-D 17 score by 49–52%, comparable to that reported in randomized clinical trials for standardized extracts (45–59%), and the percentage of responders was 50–57%. In addition, the treatment showed very good tolerability, with only 4% adverse reactions, significantly lower compared to 20–39% in the reference studies [223].

#### 4.5.7. Safety, Toxicity, and Uses

*Hypericum perforatum* L. is one of the most extensively studied medicinal plants; however, it is also among the herbal products most frequently associated with adverse reactions and clinically significant drug interactions [224]. A commonly reported adverse effect is photosensitization, manifested by erythema, pruritus, and cutaneous reactions upon exposure to ultraviolet radiation. This effect is primarily attributed to hypericin and has been consistently described in clinical studies and pharmacological reviews [209,225,226]. Gastrointestinal disturbances such as nausea, abdominal discomfort, constipation, and diarrhea are also reported. These reactions are generally mild and self-limiting, as observed in clinical trials evaluating St John’s wort in mild-to-moderate depression [227]. Central-nervous-system-related adverse effects include dizziness, headache, fatigue, and agitation. In patients with bipolar disorder, *Hypericum perforatum* L. has been associated with the induction of manic or hypomanic episodes, a clinically relevant concern documented in the psychiatric literature [225]. One of the most important safety considerations relates to drug–herb interactions. *Hypericum perforatum* L. is a potent inducer of cytochrome P450 enzymes—particularly CYP3A4—as well as P-glycoprotein (P-gp). This induction can significantly reduce plasma concentrations and therapeutic efficacy of numerous medications, including oral contraceptives, anticoagulants, antiretrovirals, immunosuppressants (e.g., cyclosporine), antiepileptics, and certain chemotherapeutic agents [209,228]. These interactions are considered clinically significant and may result in therapeutic failure.

Acute toxicity has been evaluated in experimental models using dried extracts from Hyperici herba and Hyperici flos, derived from both wild and cultivated sources. A toxicological assessment was conducted according to OECD Guideline TG 423 (Acute Toxic Class Method). The results indicated a low acute toxicity profile. Extracts of Hyperici herba (wild and cultivated) and Hyperici flos from wild sources were classified in toxicity class 5, with LD_50_ values > 5000 mg/kg. The cultivated Hyperici flos extract was also classified in toxicity class 5, with an LD_50_ of approximately 2500 mg/kg. Both intragastric and intraperitoneal administration resulted in very few animal deaths, and microscopic examination did not reveal pathological alterations in internal organs [229].

Nevertheless, concerns have been raised regarding use during pregnancy and lactation. Experimental studies in rats exposed to *Hypericum perforatum* L. extracts during lactation revealed hepatic lesions (vacuolization, lobular fibrosis, and disorganization of hepatic arrays) and renal alterations (reduced glomerular size, disappearance of Bowman’s space, and tubular hyaline degeneration) [230].

### 4.6. Tilia platyphyllos Scop., Tilia tomentosa Mnch., Tilia cordata Mill.

#### 4.6.1. General Description and Distribution

Species of the genus Tilia (*Tilia cordata* Mill., *Tilia platyphyllos* Scop., *Tilia tomentosa* Mnch.) are widespread in Romania, especially in hilly and forest areas, being traditionally used for their sedative and calming properties.

*Tilia cordata* Mill. has long been used in traditional medicine as a non-narcotic sedative for sleep disturbances and anxiety, largely attributed to the presence of flavonoids such as tiliroside. According to the European Pharmacopoeia, *Tiliae flos* (linden flower) is recognized as a medicinal plant material of therapeutic importance. It is derived from *Tilia platyphyllos* Scop., *Tilia cordata* Mill., and their natural hybrid *Tilia × vulgaris* [146]. Despite its longstanding use, reports regarding the chemical composition and bioactivity of *Tiliae flos* remain limited [231].

Per the European Pharmacopoeia (8th edition), *Tiliae flos* consists of a complete inflorescence with an attached bract. Its defining organoleptic characteristics include an aromatic odor and a sweet taste due to mucilage content [232]. Notably, the phytochemical composition of biologically active compounds in *Tiliae flos* derived from *Tilia tomentosa* or other *Tilia* species may differ significantly from the standardized pharmacopeial material, potentially influencing therapeutic activity [146]. Silver linden is less well documented phytochemically, and this is not included in the official *“Tiliae flos”* monograph. Although the European Medicines Agency (EMA) in 2011, through the Committee on Herbal Medicinal Products (HMPC), considered its inclusion in the European regulatory framework, insufficient evidence of traditional use across EU member states prevented its incorporation into the community monograph [233].

#### 4.6.2. Level of Scientific Evidence

Species in the genus *Tilia* are mainly supported by traditional use and preclinical data, with limited clinical evidence of anxiolytic effects [233].

#### 4.6.3. Phytochemical Composition

Linden flowers contain flavonoids, phenolic acids, coumarins, monoterpenes, and sesquiterpenes (Table 3) responsible for the pharmacological effects [232]. Toker et al. reported that quercetin, kaempferol, tiliroside, and astragalin—identified in *Tilia* species—are known to exert sedative and neuroprotective effects and may contribute to anxiolytic activity through enhancement of GABAergic transmission and reduction in neuronal excitability [234].

#### 4.6.4. Mechanisms of Action

The sedative and anxiolytic effects of *Tilia* species are associated with action on the central nervous system and reduction in neuronal excitability, contributing to the induction of relaxation and stress relief [233].

#### 4.6.5. Preclinical Data

Recent preclinical studies support the anxiolytic and sedative potential of species within the genus *Tilia* through centrally mediated mechanisms [235]. Allio et al. further investigated the effects of *Tilia tomentosa* Moench bud extracts on GABAergic synapses using voltage-clamp recordings and micro-electrode arrays (MEA) in primary hippocampal neuron cultures. The study demonstrated that direct postsynaptic application of bud extracts activated a chloride current resembling that induced by GABA, which was predominantly inhibited by the GABA_A_ antagonists bicuculline and picrotoxin. Additionally, evidence suggested that bud extracts may interact with both GABA_A_ receptors and benzodiazepine (BDZ) binding sites. MEA analyses revealed that low extract concentrations reduced neuronal excitability and increased network synchronization, thereby enhancing inhibitory transmission in mature hippocampal neurons [236]. An EMA report published in 2015 indicated that aqueous extracts from *Tilia cordata* Mill. flowers exhibited in vitro immunostimulatory effects, as evidenced by stimulation of lymphocyte proliferation at a concentration of 20 μg/mL. This response was comparable to that induced by specific ligands of the peripheral benzodiazepine receptor, suggesting a possible modulatory effect at this receptor site. Additionally, significant central nervous system sedative effects [237] were observed in mice following inhalation of essential oil obtained from *Tilia* spp. flowers, further supporting the plant’s traditional calming use [238]. Turrini et al. conducted a 21-day in vivo experimental study demonstrating that extracts obtained from the buds of *Tilia tomentosa* Moench reduced anxiety-like behavior in C57BL mice. The observed effects were age- and sex-dependent and were attributed primarily to the plant’s phytochemical profile, rich in phenolic compounds—particularly flavonoids such as quercetin, isoquercetin, rutin, kaempferol, and apigenin derivatives—as well as phenolic acids [56].

#### 4.6.6. Clinical Data

Clinical data are limited, with use mainly supported by tradition and phytotherapeutic monographs [233].

#### 4.6.7. Safety, Toxicity, and Uses

*Tilia* species are considered safe, being frequently used in phytotherapeutic products and dietary supplements with sedative and anxiolytic effects. Preparations derived from Tiliae species (flowers, bracts, and buds—used in gemmotherapy) are generally regarded as safe; however, the literature reports several potential adverse effects, primarily related to central nervous system and cardiovascular activity. The most frequently described adverse effect is mild-to-moderate sedation, manifested by drowsiness and reduced alertness. This effect is attributed to the presence of flavonoids and other anxiolytic compounds and is consistently mentioned in pharmacological reviews of the genus Tilia [233,239]. When co-administered with other sedative agents or central nervous system depressants (e.g., benzodiazepines, hypnotics), Tilia preparations may potentiate sedative effects, warranting caution in combined therapy [239].

At the cardiovascular level, mild decreases in blood pressure and transient palpitations have been reported, particularly in sensitive individuals or following higher doses. These effects are thought to be related to the vasodilatory and spasmolytic properties of the plant. Occasional mild allergic reactions, including skin rash and pruritus, have also been described, especially in individuals with hypersensitivity to plants within the Malvaceae/Tiliaceae family [239].

With regard to toxicity, most official assessments and phytotherapy reviews consider Tiliae species to have a favorable safety profile, largely due to the absence of strong evidence indicating systemic toxicity in healthy individuals [233]. Potential health risks are more likely to arise from contamination with heavy metals [240] or from individual allergic reactions. Ferreira T. et al. demonstrated that oral administration of *Tilia platyphyllos* Scop. extract (4.5 mg/10 mL) for 33 days in HPV16 transgenic mice was safe and well tolerated under the experimental conditions, with no significant toxicological alterations observed [241]

### 4.7. Crataegus monogyna Jacq.

#### 4.7.1. General Description and Distribution

*Crataegus monogyna* Jacq. is a widespread species in the spontaneous flora of Romania, traditionally used for its cardiotonic and sedative effects. Hawthorn (*Crataegus monogyna* Jacq.) is widely recognized for its beneficial effects on human health, particularly within the cardiovascular system, and represents a rich source of bioflavonoids with pronounced antioxidant activity. Although *Crataegus monogyna* Jacq. is traditionally regarded as one of the most valuable herbal remedies for cardiovascular disorders, emerging evidence suggests that it may also have therapeutic potential in anxiety-related conditions [80].

#### 4.7.2. Level of Scientific Evidence

*Crataegus monogyna* Jacq. is supported by phytochemical and pharmacological studies, as well as some clinical data on effects on the cardiovascular system and anxiety [156,242].

#### 4.7.3. Phytochemical Composition

The plant contains (according to Table 3) numerous flavonoids, phenolic acids, pentacyclic terpenic acids, and sterols that contribute to its pharmacological effects [156].

#### 4.7.4. Mechanisms of Action

The pharmacological effects of *Crataegus monogyna* Jacq. are associated with anti-oxidant, cardioprotective actions and modulation of the autonomic nervous system, contributing to reduction in stress and anxiety [242].

#### 4.7.5. Preclinical Data

Preclinical studies have shown that extracts obtained from the pulp and seeds of *Crataegus monogyna* Jacq., administered to mice at doses ranging from 1 to 1000 mg/kg, produced significant, dose-dependent reductions in locomotor activity and exploratory behavior in activity cage tests. These findings suggest potential utility in the management of stress, nervousness, and sleep disturbances [243]. In another experimental study, hawthorn extract reduced anxiety-like behavior induced in dark–light box paradigms at doses of 100 and 200 mg/kg [243]. Another species, *Crataegus laevigata* (Poir.) DC. (syn. *Crataegus oxyacantha*), has also been investigated for its anxiolytic potential, particularly in fixed combinations with *Eschscholzia californica* Cham. [80].

#### 4.7.6. Clinical Data

Clinical evidence remains limited but suggestive. In a double-blind, placebo-controlled study, Abbasi et al. demonstrated that fruit extract of *Crataegus monogyna* Jacq. could be used as adjunctive therapy for blood pressure control and sleep quality improvement in patients with hypertension and concomitant sleep disturbances. After eight weeks of treatment, the intervention group showed significant reductions in systolic and diastolic blood pressure compared to placebo. Furthermore, Pittsburgh Sleep Quality Index scores revealed a significant decrease in sleep disturbance severity in the hawthorn-treated group (*p* = 0.001) [244]. In a randomized, placebo-controlled clinical trial involving 264 patients with mild-to-moderate anxiety (DSM-III-R criteria), Hanus et al. reported that the fixed combination of these two plant extracts resulted in a statistically significant clinical improvement compared with placebo. Reductions in total Hamilton Anxiety Rating Scale scores and subjective anxiety assessments were significant, supporting a genuine anxiolytic effect beyond placebo [242].

#### 4.7.7. Safety, Toxicity, and Uses

When administered at therapeutic doses, hawthorn preparations are generally associated with mild adverse effects. Reported reactions include sweating, headache, mild skin eruptions, palpitations, drowsiness, agitation, and minor gastrointestinal disturbances [245]. In a large observational study including 5577 patients receiving standardized hawthorn extracts at doses ranging from 160 to 1800 mg for periods between 3 and 24 weeks, 166 patients reported adverse effects of mild-to-moderate intensity. The most frequently documented events were dizziness (*n* = 5), gastrointestinal disturbances (*n* = 24), headache (*n* = 9), migraine (*n* = 8), and palpitations (*n* = 11) [246]. The reported median lethal dose (LD_50_) values indicate that the hydroalcoholic extract obtained from hawthorn leaves and fruits has an oral toxicity of 18.5 mL/kg in mice and 33.8 mL/kg in rats. Also, for the fractions with high flavonoid content administered intravenously, the LD_50_ was estimated to be 1.56 g/kg in mice. Regarding the proanthocyanidin fraction, LD_50_ values of 130 mg/kg were determined for intraperitoneal administration and 300 mg/kg for subcutaneous administration in mice [156]. When combined with medications used in cardiovascular conditions, it can potentiate or diminish the effects of treatments used in hypertension, angina pectoris, heart failure, and heart rhythm disorders. It can also interact with beta-blockers and cardiac glycosides due to the cardiotonic and hypotensive properties that this plant possesses [156].

Several limitations should be considered when interpreting the available evidence. Although such issues are common in medicinal plant research, they manifest differently depending on the species. For instance, while *Hypericum perforatum* L. is supported by well-documented clinical data, other species such as *Galium odoratum* (L.) Scop. and *Leonurus cardiaca* L. rely predominantly on preclinical evidence. In addition, variability in phytochemical composition, extraction methods, and plant material may influence the reproducibility of results, particularly for species such as *Crataegus monogyna* Jacq. and *Tilia* spp. Therefore, future research should focus on standardized extracts and well-designed clinical trials targeting specific plant species rather than general assumptions.

The available adverse reactions and toxicity data are summarized in Table 4, separating central and peripheral toxicities. This presentation highlights that the safety profile differs significantly between the species analyzed, ranging from predominantly mild and poorly documented adverse reactions to more well-documented drug interactions and systemic toxic effects.

The pharmacokinetics and drug–plant interactions for the studied plants are briefly presented in Table 5.

The drug–herb interactions reported vary considerably depending on the species, from well-documented mechanisms to only theoretical effects. *Hypericum perforatum* L. has the highest potential for interaction, acting as a major enzyme inducer (CYP450 and P-gp), which can reduce the efficacy of essential drugs (e.g., cyclosporine, digoxin, oral contraceptives) and increase the risk of serotonin syndrome in combination with SSRIs. In contrast, for *Matricaria chamomilla* L. and *Melissa officinalis* L., interactions are less documented, being considered additive effects accompanied by sedation or possible interference with anticoagulants, requiring clinical caution. For *Leonurus cardiaca* L., Tilia spp., and *Crataegus monogyna* Jaq., interactions are predominantly pharmacodynamic (antihypertensives or digoxin), but the level of evidence is limited, and recommendations are based mainly on caution. In the case of *Galium odoratum* L., the lack of clinical data makes interactions only theoretical, being mainly associated with the presence of coumarins. Overall, these results highlight the need for a differentiated risk assessment, from strict avoidance of associations (in the case of St. John’s wort) to monitoring or caution in use for the other species.

## 5. Pharmaceutical Products, Patents, OTC Preparations, and Widely Available Dietary Supplements Containing the Selected Plant Species

In recent years, growing interest in the use of medicinal plants for the management of depression and anxiety has been reflected not only in the expanding scientific literature but also in the increasing number of patents focused on the development of functional products, nutraceuticals, dietary supplements, and herbal medicinal products. A detailed analysis of these patents provides valuable insight into current innovation trends, preferred extract combinations, proposed therapeutic applications, and formulation strategies, thereby illustrating the transition from basic research to practical and commercial implementation.

Taking these considerations into account, Table 6 presents selected patents based on the medicinal plants discussed in this review, either used individually or combined with other botanical extracts or bioactive compounds.

The selection of patents was made based on their relevance for the targeted indications, namely the reduction in symptoms associated with anxiety, stress, insomnia, and cognitive disorders. Different types of formulations and products, registered in various geographical regions (United States, China, South Korea, Russia, and Europe), were included to reflect the diversity and global interest in the valorization of these species.

Although this review focuses on species from the spontaneous flora of Romania, many of these plants are widely distributed in other regions of the world due to similar pedoclimatic conditions. Currently, there are not enough patents developed at the national level strictly focused on the use of these species for anxiety and depression. Therefore, we reviewed some examples of relevant international patents to highlight the practical applicability of these species and the way in which they are currently studied and used. It is important to emphasize that the existence of a patent does not automatically imply clinical validation of efficacy. However, patent activity clearly reflects the growing commercial and scientific interest in harnessing the therapeutic potential of medicinal plants and highlights emerging formulation strategies in phytotherapeutic approaches to neuropsychiatric disorders.

As shown in Table 6, no patents were identified involving *Galium odoratum* (L.) Scop. for anxiolytic or antidepressant indications. However, a detailed patent analysis revealed its inclusion in various electronic aerosol-generating devices intended for the controlled inhalation of nicotine or other substances. In these patents, sweet woodruff is mentioned as a potential aromatic botanical ingredient, alongside numerous other plant species [275], primarily to enhance the flavor and aroma of tobacco substitutes [276]. Additionally, in oral formulations containing psychoactive mushroom-derived compounds, *Galium odoratum* (L.) Scop. is incorporated to modify sensory perception, improve taste and aroma, and increase treatment compliance [277].

With regard to *Melissa officinalis* L., a Chinese patent describes its inclusion as a pharmacologically active component in tablet formulations intended for the prevention and treatment of diseases associated with angiogenesis and matrix metalloproteinase (MMP) activity, with potential applications in obesity, cancer, and chronic inflammation [278]. In South Korea, in 2022, extracts obtained from lemon balm leaves, together with rosmarinic acid, were included as active ingredients in cosmetic patents. These components are reported to increase the expression of the sodium–hydrogen exchanger 1 (NHE1), a factor involved in the regulation of skin pH, thereby contributing to the maintenance of a mildly acidic cutaneous environment [279]. Furthermore, ongoing research increasingly explores the relationship between phytotherapy and the gut–brain axis, an area of growing global interest, offering potential non-pharmacological strategies for conditions such as chronic stress, anxiety, and depression.

Given the continuously growing interest in complementary therapies, particularly in the management of anxiety and depression, phytotherapeutic preparations and dietary supplements play an important role in current clinical practice. A wide range of plant-based products is available on the Romanian market, commonly used as adjuvant therapies in mild-to-moderate affective disorders.

In this context, Table 7 presents the main phytotherapeutic preparations and some over-the-counter (OTC) products sold in pharmacies in Romania, highlighting the composition, mode of administration, and declared therapeutic activity. The inclusion of these data aims to correlate the scientific information presented in the review with existing practical applications, providing an image of the current level of valorization of these species at the national level.

As highlighted in Table 7, the analyzed products are classified either as dietary supplements or over-the-counter (OTC) medicines and are generally considered safe, as they tend to produce fewer adverse effects compared with conventional pharmacotherapy, particularly tricyclic antidepressants (TCAs), which are associated with anticholinergic symptoms, sexual dysfunction, insomnia, and withdrawal-related complications [4]. The presented data indicate that most preparations contain plant extracts with anxiolytic and sedative potential, such as *Hypericum perforatum* L., *Melissa officinalis* L., and *Leonurus cardiaca* L. These extracts are frequently combined with minerals (especially magnesium), omega-3 fatty acids, or other compounds with adaptogenic potential to support stress resilience and emotional balance.

However, classification as a dietary supplement entails a regulatory framework distinct from that applied to OTC medicinal products, particularly regarding requirements for standardization, demonstration of clinical efficacy, and rigorous evaluation of the risk–benefit profile. In contrast, products classified as OTC medicines—such as certain *Hypericum perforatum* L.-based preparations—are subject to stricter quality control criteria, extract standardization, and documentation of traditional or clinical use in mild-to-moderate depression.

It is important to emphasize that the existence of a patent or a commercially available product does not automatically equate to proven clinical efficacy or long-term safety. Independent quality assessments and post-marketing surveillance studies are warranted to verify compliance with declared specifications and to further characterize the safety profile for consumers.

## 6. Study Limitations

The present work is a narrative review and, as such, presents methodological limitations inherent to this type of analysis. The study selection did not follow a standardized systematic protocol (e.g., PRISMA), which may introduce selection bias and the potential omission of relevant studies. Furthermore, a formal risk-of-bias assessment (e.g., RoB tools for clinical or preclinical studies) was not performed; therefore, it was not possible to stratify the strength of the conclusions according to the methodological quality of the included studies.

The synthesized data vary in terms of study design (in vitro experiments, animal models, small-scale clinical trials), plant material characteristics (species, plant part used, extraction methods), and investigated endpoints. This variability limits the direct comparability of results and precludes the formulation of quantitative conclusions or the performance of a meta-analysis.

For most of the analyzed species, evidence regarding anxiolytic and antidepressant effects derives predominantly from preclinical studies, whereas clinical trials remain limited, often characterized by small sample sizes and short follow-up periods. Additionally, pharmacokinetic and pharmacodynamic data, as well as longitudinal safety evaluations, were not systematically included.

A major limitation is the variability in phytochemical composition, particularly in species such as *Crataegus monogyna* Jacq. and *Tilia* spp., where differences in extraction methods and plant parts may influence biological activity.

There is also the possibility of publication bias and language bias. Information regarding commercial products and patents reflects the status available at the time of manuscript preparation and may change over time.

While the analysis incorporates species occurring in the Romanian spontaneous flora and considers products available on the national market, these plants are widely distributed and utilized across multiple regions. Therefore, the conclusions should be interpreted within a broader international context, taking into account variations in regulatory frameworks, market availability, and patterns of use rather than being viewed as strictly region-specific.

## 7. Future Perspectives

Future research should focus on the development of standardized protocols for the preparation and characterization of plant extracts, taking into account phytochemical variability determined by species, subspecies, geographical origin, plant part, and processing methods. Such standardization is essential to ensure the reproducibility of findings and to facilitate comparisons between preclinical and clinical studies.

Regarding mechanisms of action, future investigations should aim to further elucidate the molecular targets involved in anxiety and depressive disorders, including the GABAergic system, monoaminergic neurotransmission, the hypothalamic–pituitary–adrenal (HPA) axis, neuroinflammatory processes, and neuroplasticity-related mechanisms. Integrated experimental approaches combining pharmacological, molecular, and behavioral methodologies may help clarify the relationship between phytochemical composition and observed biological effects.

From a future perspective, advancing the clinical relevance of the analyzed plant species requires a more coherent integration of existing pharmacological, preclinical, and emerging clinical data. Particular attention should be given to species for which the current evidence remains predominantly experimental, by promoting research strategies that facilitate the translation of mechanistic findings into clinically meaningful outcomes.

In addition, exploring their role as adjunctive therapies alongside conventional treatments represents a promising direction, especially in the context of multifactorial disorders such as anxiety and depression. Such approaches may contribute to optimizing therapeutic responses, improving tolerability, and supporting the gradual integration of phytotherapy into evidence-informed clinical practice.

## 8. Conclusions

The present review highlights that medicinal plants from the spontaneous flora of Romania analyzed in this study exhibit variable potential in the management of anxiety and depressive symptoms. Among them, *Hypericum perforatum* L. and *Melissa officinalis* L. are supported by the most consistent clinical evidence, whereas for other species—such as *Galium odoratum* (L.) Scop., *Leonurus cardiaca* L., *Tilia* spp., and *Crataegus monogyna* Jacq.—the anxiolytic and antidepressant effects are primarily supported by preclinical data and traditional use.

Comparative analysis of their mechanisms of action suggests that the therapeutic effects of these plants are mediated through a combination of biological pathways, including modulation of GABAergic and monoaminergic neurotransmission, attenuation of neuroinflammation, regulation of the hypothalamic–pituitary–adrenal (HPA) axis, and support of neuroplasticity-related processes. The diversity of these mechanisms underscores the need for a differentiated and cautious approach in the use of phytotherapy for anxiety–depressive disorders.

This work emphasizes the contextual interpretation of phytochemical and pharmacological data by integrating evidence derived from globally studied species with their documented occurrence, traditional use, and current availability on the Romanian market. Rather than reiterating widely acknowledged limitations in medicinal plant research, the present review highlights how these species—although not geographically restricted to Romania—are embedded in the local ethnopharmacological framework and commercial landscape.

In this context, the analysis focuses on bridging global scientific evidence with region-specific factors, including patterns of use, market presence, and ecological distribution within Romania. This approach aims to provide a more coherent perspective that connects internationally generated data with its practical relevance at the national level, thereby supporting a more informed evaluation of their therapeutic potential.

By aligning international research data with region-specific patterns of use and availability, this study provides a context-oriented perspective that may support future research directions, phytotherapeutic applications, and product development in both European and broader international settings.

## Figures and Tables

**Table 1:** Taxonomic classification of some plants from the spontaneous flora of Romania used in depression and anxiety.

Plant Species	Popular Name	Genus	Subfamily	Family	Order	Subclass	Ref.
*Matricaria chamomilla* L.	Chamomile	Matricaria	Asteroideae	Asteraceae	Asterales	Asteridae	[68]
*Galium odoratum* (L.) Scop.	Sweet woodruff	Asperula	Rubioideae	Rubiaceae	Gentianales		
*Melissa officinalis* L.	Melissa officinalis, Lemon balm	Melissa	Nepetoideae	Lamiaceae	Lamiales		
*Leonurus cardiaca* L.	Motherwort	Leonurus	Lamioideae	Lamiaceae	Lamiales		
*Hypericum perforatum* L.	St. John’s Wort	Hypericum	Hypericoideae	Hypericaceae	Theales	Dileniidae	
*Tilia platyphyllos* Scop.	Large-leaved linden	Tilia	Tilioideae	Tiliaceae	Malvales		
*Tilia tomentosa* Mnch.	Silver linden	Tilia	Tilioideae	Tiliaceae	Malvales		
*Tilia cordata* Mill.	Brimstone linden	Tilia	Tilioideae	Tiliaceae	Malvales		
*Crataegus monogyna* Jacq.	Hawthorn	Crataegus	Maloidae	Rosaceae	Rosales	Rosidae	

**Table 2:** Botanical characteristics relevant for the identification and authentication of the analyzed species.

Species	Characteristics		Refs.
*Matricaria chamomilla* L.	Stem	Erect and branched, up to 80 cm	[68,69]
	Leaves	Long and narrow, bi or tripinnate sected, with narrow lines, alternately arranged in number of 16–40	
	Flowers	Grouped in pedunculated inflorescences that present heterogamous, conical anthodes located at the end, and, on the outside, they are surrounded by an envelope made up of two rows of ovate and green leaflets, with white and membranous edges The female, marginal flowers are white and concentrically arranged in the early phase of flowering The tubulous, hermaphroditic, central flowers are golden-yellow in color	
	Fruit	Slightly curved, yellowish-brown achene, with between 3 and 5 whitish longitudinal veins	
*Galium odoratum* (L.) Scop.	Leaves	Green, glossy, lanceolate or elliptical, glabrous The lower ones are arranged in false swirls of five to seven leaves, while the upper leaves are arranged in swirls of eight, narrowing at the base	[70,71,72,73]
	Stem	Creeping	
	Flowers	Small (4–7 mm), white, stellate, grouped in delicate inflorescences, called cymes	
*Melissa officinalis* L.	Stem	Erect, branched, and has a tetragonal cross-section, being generally glabrous	[74,75]
	Leaves	Arranged in pairs, it has an oval petiole and limb The edge of the leaf is crenate-toothed, with a cordate base and a wedge-shaped upper part The surface of the blade is rough and deeply ribbed, subglabrous, sometimes showing glandular trichomes and punctiform glands on the abaxial side	
	Flowers	It has two stamens and four lobed ovaries that form between 1 and 4 nuts and are grouped in inflorescences consisting of small buds, with 4 to 12 flowers, white or pale pink in color, which bloom during the summer	
*Leonurus cardiaca* L.	Flowers	Bilabiate and light pink in color, grouped in large, elongated, terminally located inflorescences	[76,77]
	Stem	Aerial growth from rhizomes	
	Leaves	Lobes covered with bristles	
*Hypericum perforatum* L.	Leaves	Shortly petiolate, with a maximum length of 1 mm and of the same color on both sides, sessile and opposite Have an elongated, oval, or elliptical shape	[68,78,79]
	Flowers	5 yellow petals on the surface of which numerous black glands are present and are arranged in branched tops Five short, whole, and imbricate sepals, upper ovary, capsicular with three styles, and many stamens arranged in bundles of three	
*Tilia platyphyllos* Scop.	Leaves	Large (6–12 cm), covered by whitish bristles on both sides, more visible on the back	[68,78,79]
	Flowers	Hermaphrodites arranged in hanging crowns, and the bract is shorter than the inflorescence	
	Fruits	Globular, up to 10 mm, with a pointed tip and five obvious ribs	
	Flowering	In June	
*Tilia tomentosa* Mnch.	Leaves	5–10 cm, with a dark green face and a gray back, covered with starry bristles, which give the leaf a silvery appearance	[68,78,79]
	Flowers	Hermaphrodites, 5–10 each, arranged in drooping CILs, shorter than the hairy bract	
	Fruits	Oval, tomentose, 5–7 mm, smooth or slightly ribbed	
	Flowering	In July	
*Tilia cordata* Mill.	Leaves	Small (3–6 cm), with tufts of rusty bristles at the armpit of the ribs	[68,78,79]
	Flowers	Numerous (3–16), hermaphroditic, arranged in horizontal ridges	
	Fruits	Small, globular, and glabrous, with a smooth or weakly edged surface	
	Flowering	At the beginning of summer, in the months of June–July	
*Crataegus monogyna* Jacq.	Bark	Grayish-brown, smooth, with superficial longitudinal cracks	[68,80]
	Leaves	Simple, rhombic-ovate, with straight or toothed edges, with deep lobes, dark green on the upper part and light green on the underside	
	Flowers	White or pink in color and arranged in erect corymbs, the petals are round, longer than the sepals and contain a single ovary, and each stamina is composed of 5–25 anthers. The flower buds are protected by a greenish calyx and consist of 5 sepals, and the corolla consists of 5 petals	
	Fruits	An oblong pseudodrupe bearing 1–5 pyrenes resembling those of plums; the fruits are ovoid, bright red, and fleshy, appearing in late summer to autumn	

**Table 3:** Secondary metabolites and their mechanisms of action relevant to depression and anxiety identified in the studied plants.

Plant Species	Commonly Used Parts	Secondary Metabolites	Chemical Nature	Mechanism of Action	Refs.
*Matricaria chamomilla* L.	flower	Stigmasterol	Sterols	In vivo studies have shown that at doses of 0.5–3.0 mg/kg it can positively modulate GABA receptors.	[6,85,86,87]
		α-bisabolol	Sesqviterpenes	After intraperitoneal administration, at doses of 0.5, 1, 2, 5 and 10 mg/kg, in mice, it may exhibit CNS depressant activity, due to interactions with GABAA at receptor subtypes that mediate the effects of benzodiazepines.	[6,85,86,88]
		farnesol	Sesqviterpenes	In the Murine model, performed on rats, farnesol exhibited anxiolytic, anti-nociceptive, and depressive effects.	[6,85,86,89]
		geraniol	Monoterpenic alcohols	In mice exposed to CUMS, after 3 weeks of treatment, the following was observed: reduction in neuroinflammation, particularly by decreasing IL-1β levels, inhibiting the NF-κB pathway, and regulating the NLRP3 inflammasome.	[6,85,86,90]
		borneol	Monoterpenic alcohols	In depressed rats, GRg1/GO-PEG-BO improved stress-induced anhedonia, despair, and anxiety and completely alleviated depressive symptoms.	[6,85,86,91]
		chemazulene	Azulene	Molecular docking simulations suggest that it may interact with targets involved in inflammation, apoptosis, and energy metabolism. Binding affinity for IL-6, IFN-γ and caspase-3 indicates a potential anti-inflammatory and neuroprotective effect, while interaction with GLUT1, HK1, and LDHA suggests a possible involvement in the regulation of cerebral energy metabolism, which is frequently impaired in mood disorders.	[6,47,85,86]
		esculetin	Coumarins	It ameliorated LPS-induced neuroinflammation by reducing brain levels of IL-1β, IL-6, and TNF-α, as well as oxidative stress and plasma corticosterone levels, in mice.	[6,85,86,92]
		scopoletin	Coumarins	It exerts anxiolytic and antidepressant effects by reducing neuroinflammation, mediated by inhibition of the NF-κB pathway and activation of Sirt1, leading to a decrease in proinflammatory cytokines and an improvement in pathological behaviors.	[6,85,86,93]
		umbelliferone	Coumarins	It has demonstrated effects on the serotoninergic system by increasing 5-HT levels and reducing its degradation, evidenced by a decrease in the 5-HIAA/5-HT ratio.	[6,85,86,94]
		chlorogenic acid	Phenolic acids	By reducing neuroinflammation and oxidative stress, as well as by inhibiting the NLRP3 inflammasome, it exhibits antidepressant effects. These effects are mediated by activation of the PI3K/Akt/Nrf2 pathway, which contributes to increasing the cellular antioxidant response.	[6,85,86,95]
		caffeic acid	Phenolic acids	Through epigenetic mechanisms, regulating DNA methylation, and the expression of genes involved in neuronal function, such as BDNF and COMT, it can exert antidepressant effects in mice. These effects are mediated by modifying the activity of enzymes (DNA methyltransferases and Ten-eleven translocation enzymes) and adjusting the 5-methylcytosine/5-hydroxymethylcytosine ratio at the level of promoter regions, contributing to the normalization of gene expression.	[6,85,86,96]
		apigenin	Flavonoids	It exerts antidepressant effects (induced by LPS) on mice, by inhibiting neuroinflammation, mediated by suppressing NF-κB activation and reducing iNOS and COX-2 expression, leading to a decrease in proinflammatory cytokines.	[6,85,86,97]
		luteolin	Flavonoids	By reducing neuroinflammation, improving synaptic plasticity (evidenced by increased synapsin), and increasing serotonin and noradrenaline levels, it exerts antidepressant effects in mice exhibiting noise-induced depression.	[6,85,86,98]
		quercetin	Flavonoids	By inhibiting neuronal apoptosis and activating the ERK/Nrf2 pathway, it contributes to increasing antioxidant mechanisms and improving neuronal function in mice with CUMS-induced depression.	[6,85,86,99]
		rutin	Flavonoids	It increased levels of serotonin, norepinephrine, and dopamine in cortical and hippocampal regions in rats with reserpine-induced depression. In another study, it effectively rescued CUMS-induced behavioral deficits by reducing depression, anxiety, and improving cognition and locomotor and muscle coordination abilities in mice.	[6,85,86,100,101]
		kaempferol	Flavonoids	It exerts significant antidepressant effects, mediated by reducing oxidative stress and neuroinflammation, as well as by activating the AKT/β-catenin signaling pathway.	[6,85,86,102]
		naringenin	Flavonoids	It produced behavioral modification in BALB/c mice with neuroinflammation induced by olfactory bulbectomy—oxidative stress. It also restored the levels of corticosterone in serum and antioxidant enzymes (Catalase, SOD, GSH), nitrite, and MDA in the cerebral cortex and hippocampus, demonstrating its anti-stress and antioxidant properties. It significantly reduced the elevated levels of proinflammatory cytokines (IL-1β, IL-6, TNF-α and NF-ҝβ), increased neurotrophic growth factor, as well as BDNF, and reversed the altered levels of tryptophan, serotonin, 5-hydroxyindole, acetic acid, and kynurenine in the hippocampus and cortex.	[6,85,86,103]
*Galium odoratum* (L.) Scop.	aerial part of the plant	coumarin	Coumarin	Following forced swimming and tail suspension tests performed on mice, it was observed that this compound exhibits antidepressant effects.	[75,104,105,106,107,108]
	aerial part of the plant and rhizome	asperuloside	Iridoids	In the tests, it was observed that induced depression in rats was improved, as evidenced by an increase in the number of entries into the central zone, a decrease in immobility time, and an increase in swimming time, sucrose preference, and body weight. It also activated the Wnt3α/glycogen synthase kinase 3β (GSK-3β)/β-catenin signaling pathway in vivo.	[109]
		monotropein	Iridoids	Significantly improves LPS-induced depression-like symptoms in mice, possibly by inhibiting the cGAS/STING signaling pathway.	[110]
		aucubin	Iridoids	Ameliorates depression by promoting intranuclear expression of the glucocorticoid receptor (GR) and inhibiting nuclear factor kappa B (NF-κB)-mediated inflammatory activation, thereby reducing cellular pyroptosis, in mice with CUMS-induced depression.	[111]
		p-coumaric acid	Phenolic acids	It improved LPS-induced despair-related behavioral symptoms in the FST, TST, and SST. It also prevented the increase in inflammatory cytokines in the hippocampus (COX-2 and TNF-α) and the reduction in BDNF.	[94]
		ferulic acid	Phenolic acids	It improved CUMS-induced depressive behaviors in the sucrose preference test and forced swim test. It increased BDNF, PSD95, and synapsin I levels in the prefrontal cortex and hippocampus. Neurochemical tests suggested that ferulic acid increased levels of monoamine neurotransmitters in the hippocampus and frontal cortex of mice. Increased levels of serotonin and norepinephrine were also observed in the hypothalamus after higher doses of ferulic acid treatment. Further study suggested that monoamine oxidase A (MAO-A) activity was inhibited in the frontal cortex and hippocampus upon treatment with 40 and 80 mg/kg ferulic acid, while MAO-B activity did not change significantly.	[112,113]
	leaf	quercitrin	Flavonoids	Levels of brain monoamines (5-HT and dopamine) and their metabolites (5-hydroxy-3-indoleacetic acid, 3,4-dihydroxyphenylacetic acid, and homovanillic acid) were decreased after quercitrin treatment. These data suggest that the anxiolytic effects of quercitrin may be mediated by the 5-HT1A receptor.	[114]
		naringin	Flavonoids	In mice with CORT-induced depression, naringin treatment promoted neuronal differentiation and maturation of NSPCs for hippocampal neurogenesis. The forced swim test, tail suspension test, and open-field test confirmed the antidepressant and anxiolytic effects of naringin. Co-treatment with temozolomide, a neurogenic inhibitor, abolished these antidepressant and anxiolytic effects. Meanwhile, naringin treatment increased the phosphorylation of cAMP response element binding protein (CREB) but had no effect on the expression of brain-derived neurotrophic factor and TrkB phosphorylation in the hippocampus of CORT-induced depressed mice. Co-treatment with the CREB inhibitor 666-15, instead of the TrkB inhibitor Cyc-B, abolished the neurogenesis-promoting and antidepressant effects of naringin.	[115]
		hesperidin	Flavonoids	It ameliorated depressive behaviors in CUMS rats. In addition, it decreased IL-1β, IL-6, TNF-α, NLRP3, caspase-1, and ASC levels in PFC and microglia.	[116]
		cynaroside	Flavonoids	In vivo studies have shown that it can improve states of anxiety, despair, and anhedonia in CUMS mice and that it can reduce microglial activation in the hippocampus. This may suggest that cynaroside, by inhibiting the polarization of microglial cells into the M1 phenotype and reducing the levels of inflammation and ferroptosis, exhibits an antidepressant effect.	[117]
*Melissa officinalis* L.	essential oil obtained from leaves	citronellal	Monoterpenes	Behavioral analyses (open-field, hole-cross, swing, and light–dark tests) showed that it can significantly and dose-dependently reduce locomotor activity, that it can potentiate the anxiolytic effects induced by diazepam, and that it can counteract the effects of flumazenil, suggesting GABAergic modulation, with binding affinities (–4.7 and –4.0 kcal/mol) to the α2 and α3 subunits of GABAA.	[118,119,120]
		caryophyllene	Sesqviterpenes	In adult mice, it induced improvement in all the parameters evaluated in the plus maze test. It could also significantly increase the time spent in the center of the arena in the open-field test, without modifying general motor activity; it could reduce the number of buried balls and the time spent digging in the MBT, suggesting an anti-compulsive effect.	[121]
	aerial part of the plant	rosmarinic acid	Phenolic acids	By significantly reducing immobility time in the TST in mice, by downregulating Mkp-1, and by upregulating BDNF, Th, and Pc in the limbic system, it may exhibit antidepressant effects. Furthermore, in the mouse brain, this compound significantly reduced serum corticosterone levels and increased dopamine levels in the limbic system.	[122]
		protocatechuic acid	Phenolic acids	It significantly attenuated behavioral and neurobiochemical changes in olfactory bulbectomized rats, such that its activity was similar to that of an antidepressant, being mediated largely by modulation of neurotransmitters and endocrine and immunological systems, especially through improvements in BDNF, 5-HT, DA, and NE and reduction in MDA, IL-6, and TNF-α in the hippocampus and cerebral cortex.	[123]
		ursolic acid	Pentacyclic triterpenic acid	Microglial activation, increased levels of TNF-α, and IDO1 mRNA expression were attenuated by ursolic and oleanolic acid administration. In addition, the decreased levels of synaptophysin expression were increased by both acids, whereas the decreased level of PSD-95 was upregulated by oleanolic acid administration.	[124]
		oleanolic acid	Pentacyclic triterpenic acid	Female mice with maternal separation-induced depression exhibited stronger depression-like behaviors than males, and oleanolic acid improved several depression-like behaviors, while ursolic acid only improved anxiety-like behaviors in mice.	[124]
*Leonurus cardiaca* L.	leaves, flower, stem	rutin	Flavonoids	-	[125]
		caffeic acid	Phenolic acid	-	[125]
	aerial part of the plant	ferulic acid	Phenolic acids	-	[125]
		chlorogenic acid	Phenolic acids	-	[77,126]
		chicoric acid	Phenolic acids	The antidepressant activity may be attributed to its modulatory effect on nor-adrenaline, dopamine, and 5-hydroxytryptamine, as demonstrated by their quantification in chronically stressed mice treated with chicoric acid.	[77,126,127]
		stachydrine	Betaine alkaloid	Molecular docking analyses suggest that stachydrine significantly attenuated LPS-induced upregulation of NF-κB and NLRP3, indicating suppression of inflammasome-mediated neuroinflammatory pathways. These results suggest that stachydrine exerts neuroprotective and antidepressant effects by modulating inflammatory signaling.	[77,126,128]
		verbascoside	Phenylethanoid glycosides	It can modulate monoamine neurotransmitter levels, inhibit hypothalamic–pituitary–adrenal (HPA) axis hyperfunction, and promote neuroprotection. It also promotes dopamine (DA) biosynthesis through the expression of tyrosine hydroxylase mRNA and protein, upregulates the expression of 5-hydroxytryptamine receptor 1B, prominent protein, microtubule-associated protein 2 (MAP2), heme oxygenase-1 (HO-1), SQSTM1, recombinant autophagy-associated protein 5 (ATG5), and Beclin-1, and downregulates the expression of caspase-3 and α-synuclein, thereby exerting antidepressant effects.	[77,126,129]
		leonurine	Alkaloid	It effectively restored the levels of 5-hydroxytryptamine, noradrenaline and dopamine in the hippocampus and prefrontal cortex of chronic mild stress mice, accompanied by the amelioration of hippocampal neuronal damage. It significantly inhibited the production of proinflammatory cytokines interleukin-1β, interleukin-6, and TNF-α and suppressed the nuclear factor kappa B signaling pathway.	[10,77,126]
		caryophyllene	Sesqviterpenes	-	[77,126]
	leaf	α-pinene	Monoterpenes	It significantly improved behavioral outcomes, reduced corticosterone levels, decreased MDA levels, and increased GPx activity in stressed rats, with effects comparable to imipramine. These findings suggest that alpha-pinene may improve behavioral performance through antioxidant mechanisms and by modulating HPA axis activity, as evidenced by decreased corticosterone levels.	[77,126,130]
		linalool	Monoterpenes	The mechanisms through which linalool improves memory loss and behavioral alterations in sleep-deprived mice appeared to be through an increase in serotonin levels. Linalool significantly ameliorated the spatial and learning memory deficits and stress activity observed in sleep-deprived animals. Moreover, linalool led to serotonin release and cortisol level reduction. Our findings suggest that linalool has beneficial effects on the memory loss and behavioral alterations induced by REM-sleep deprivation through the regulation of serotonin levels.	[77,126,131]
		limonene	Monoterpenes	Limonene also reduces serum and brain nitrite levels and reduces the expression of IL-1*β* and TNF-*α* in the hippocampus. The genome-wide m6A assay and MeRIP-qPCR results revealed that the m6A modifications of Akt3, Ntrk2, Braf, and Kidins220 mRNA were significantly altered in the hippocampi of UCMS mice after stress stimulation and were reversed by hypericin treatment.	[77,126,132]
		ursolic acid	Pentacyclic triterpenic acid	-	[126]
*Hypericum perforatum* L.	leaf and stem	hypericin	Naphthodianthrones	Molecular pharmacology experiments showed that hypericin treatment upregulated the expression of m6A-modifying enzymes METTL3 and WTAP in the hippocampi of UCMS mice.	[79,133,134,135]
		pseudohypericin	Naphthodianthrones	CRF appears to be a major factor in the regulation of hypothalamic–pituitary–adrenal activity through the activation of CRF1 receptors. Following a study conducted on recombinant Chinese ovarian cells stimulated with CRF, the effect of certain constituents present in St. John’s wort on cAMP formation was measured, and it was observed that, of all the constituents, only pseudohypericin selectively antagonized CRF (KB 0.76 μM).	[79,134,135,136]
	leaf, stem, flower	hyperforin	Phlorogluninols	A randomized, double-blind, placebo-controlled study conducted on 147 patients with mild-to-moderate depression (according to DSM-IV) evaluated the efficacy of two extracts of hypericum perforatum differing in hyperforin content (0.5% vs. 5%), administered for 42 days. Efficacy was assessed by the HAMD-17, D-S and CGI scales. The results showed a significantly greater reduction in HAMD score in the group treated with the extract containing 5% hyperforin (−10.3 ± 4.6) compared to placebo (*p* = 0.004), while the extract with 0.5% hyperforin had a similar effect to placebo.	[137]
		kaempferol	Flavonoids	-	[79,134,135]
		luteolin	Flavonoids	-	[79,134,135]
		myricetin	Flavonoids	It specifically reduced immobility time in mice exposed to chronic stress, improved hippocampal glutathione peroxidase (GSH-PX) activities, reduced plasma corticosterone levels, and normalized low BDNF levels in stressed mice.	[138]
		quercetin	Flavonoids	-	[79,134,135]
		rutin	Flavonoids	-	[79,134,135]
		quercitrin	Flavonoids	-	[79,134,135]
		p-cumaric acid	Phenolic acids	-	[79,134,135]
		caffeic acid	Phenolic acids	-	[79,134,135]
		ferulic acid	Phenolic acids	-	[79,134,135]
		gentisic acid	Phenolic acids	It demonstrated significant antidepressant effects in chronic stress models in mice, evidenced by reduced immobility time and amelioration of anhedonia. The antidepressant effect was shown to be multifactorial and involved inhibition of monoamine oxidase A (MAO-A), which increased the availability of monoamines (e.g., serotonin, dopamine); an antioxidant effect manifested by decreasing oxidative stress (decreasing malondialdehyde and increasing catalase and GSH concentration); an anti-inflammatory effect manifested by reducing TNF-α and nitrites; and modulation of the HPA axis by decreasing corticosterone levels.	[139]
		shikimic acid	Phenolic acids	Inhibits the production of pro-inflammatory mediators and ROS in LPS-induced BV2 cells. Mechanistic studies demonstrated that shikimic acid suppresses neuroinflammation by activating the AKT/Nrf2 pathway and inhibiting the NF-κB pathway. Further in vivo studies confirmed that shikimic acid ameliorates the neurological damage and behavioral deficits caused by LPS injection in mice.	[140]
		chlorogenic acid	Phenolic acids	-	[79,134,135]
		isovalerianic acid	Carboxylicic acids	In a study that targeted a murine model of CRS in mice, the effect of exogenously added sodium isovalerate on mice with CRS was investigated. After feeding the mice with sodium isovalerate, an improvement in depressive behavior and an increase in 5-HT secretion in the brain and hypothalamus were observed.	[141]
		α-pinene	Monoterpenes	-	[79,134,135]
*Tilia tomentosa* Mnch.	flower	β-caryophyllene	Sesqviterpenes	-	[142]
		ferulic acid	Phenolic acids	-	[142]
		vanillic acid	Phenolic acids	In an experimental model of LPS-induced stress in rats, this compound improved anxious and depressive behaviors. The mechanism of action is mainly related to its antioxidant activity, manifested by the increase in antioxidant enzymes (SOD and GPx) and the reduction in lipid peroxidation (MDA), which leads to the reduction in oxidative stress in the brain. Thus, vanillic acid exerts a neuroprotective effect and contributes to the restoration of the redox balance involved in the pathogenesis of depressive and anxiety disorders.	[143]
		myricetin	Flavonoids	-	[142]
		rutin	Flavonoids	-	[142]
		hyperoside	Flavonoids	The main active compound of *Hypericum perforatum* demonstrated antidepressant effects in models of chronic stress, significantly improving depressive behaviors. The mechanism of action is mainly associated with anti-inflammatory effects, by inhibiting the NLRP1 inflammasome, as well as modulating the CXCL1/CXCR2/BDNF signaling pathway, which contributes to reducing neuroinflammation and stimulating neuroplasticity. In addition, the involvement of targets such as TNF-α, IL-2, and TLR4 suggests complex regulation of the immune response responsible for the antidepressant effect.	[144]
		kaempferol	Flavonoids	-	[142]
		catechin	Flavonoids	It demonstrated antidepressant and anxiolytic effects in a stress model induced by chronic corticosterone administration, improving depressive and anxious behaviors. The mechanism of action is mainly associated with the modulation of the central noradrenergic system, evidenced by the reduction in TH expression in the locus coeruleus, suggesting a regulation of noradrenaline synthesis. In addition, it contributed to the normalization of stress-induced HPA axis disorders, indicating an integrated effect on the neuroendocrine response and neurotransmission involved in depression and anxiety.	[145]
*Tilia platyphyllos* Scop., *Tilia cordata* Mill.	flower	quercetin	Flavonoids	-	[146,147,148]
		rutin	Flavonoids	-	[146,147,148]
		hyperoside	Flavonoids	-	[146,147,148]
		quercitrin	Flavonoids	-	[146,147,148]
		kaempferol	Flavonoids	-	[146,147,148]
		astragalin	Flavonoids	It has antidepressant effects demonstrated in a chronic stress model, where it improved depressive behaviors and reduced microglial activation at the hippocampal level. The mechanism of action is centered on the activation of the SIRT1 pathway, which determines the inhibition of NF-κB signaling and, consequently, the deactivation of the NLRP3 inflammasome (with the reduction in caspase-1, IL-1β, and gasdermin D). Through these effects, astragalin reduces neuroinflammation and pyroptosis processes, contributing to its antidepressant effect.	[149]
		tiliroside	Flavonoids	It demonstrated significant antidepressant effects in behavioral models (FST, TST), with a dose-dependent response. Although the exact mechanism is not fully elucidated in this study, the data suggest that its effect is associated with the modulation of central neurotransmission, most likely through interaction with monoaminergic systems (serotoninergic and/or noradrenergic), similar to classical antidepressants. In addition, the observed sedative effect indicates possible action on the central nervous system through GABAergic mechanisms or by reducing neuronal excitability, contributing to the overall antidepressant effect.	[150]
		farnesol	Sesqviterpenes	-	[146,147,148]
		linalool	Monoterpenes	-	[146,147,148]
		geraniol	Monoterpenes	-	[146,147,148]
		terpineol	Monoterpenes	It demonstrated antidepressant effects in experimental models of LPS-induced depression, significantly reducing depressive-like behaviors. The mechanism of action is mainly associated with the modulation of the endocannabinoid and dopaminergic systems, through interaction with CB1 and CB2 receptors, as well as with dopaminergic D2 receptors, an aspect supported both by molecular docking studies and by blocking effects by specific antagonists. The absence of involvement of the adrenergic and adenosinergic systems suggests selective action on these pathways. Overall, terpineol exerts antidepressant effects by regulating dopaminergic neurotransmission and endocannabinoid signaling, contributing to the improvement of the stress response and depressive symptoms.	[151]
		citral	Monoterpenes	Citral formulated in nanophytosomes demonstrated antidepressant effects in a chronic stress model, improving depressive behaviors and associated neurobiological changes. The mechanism of action is complex and mainly involves reducing neuroinflammation (by decreasing TNF-α and IL-6), combating oxidative stress (by increasing antioxidant capacity) and restoring neuroplasticity by increasing BDNF expression. In addition, citral contributes to the normalization of the stress response by decreasing cortisol levels, indicating a possible modulation of the HPA axis.	[152]
		citronellal	Monoterpenes	-	[146,147,148]
		eugenol	Monoterpenes	It is a bioactive compound with antidepressant and neuroprotective effects, acting through multiple mechanisms in the central nervous system. The mechanism of action involves the inhibition of MAO-A, which leads to increased levels of monoamines (serotonin and dopamine), as well as the stimulation of BDNF expression, contributing to the improvement of neuroplasticity. In addition, eugenol exerts antioxidant and anti-inflammatory effects and protects neurons by reducing excessive Ca^2+^ influx induced by β-amyloid peptides, thus preventing neuronal death. These integrated actions support its antidepressant and neuroprotective effects.	[153]
		limonene	Monoterpenes	-	[146,147,148]
		α-pinene	Monoterpenes	-	[146,147,148]
		fraxetin	Coumarins	It demonstrated antidepressant and anxiolytic effects in a chronic stress model, improving altered affective and cognitive behaviors. The mechanism of action mainly involves modulation of the HPA axis, by reducing elevated corticosterone levels, as well as restoring monoaminergic neurotransmission, evidenced by increased serotonin in the cortex, hippocampus, and striatum, increased noradrenaline in the striatum, and normalization of dopamine in the frontal cortex. These effects suggest that fraxetin acts by regulating the stress response and rebalancing the neurotransmitter systems involved in depression and anxiety.	[154]
		esculetin	Coumarins	-	[146,147,148]
		caffeic acid	Phenolic acids	-	[146,147,148]
		chlorogenic acid	Phenolic acids	-	[146,147,148]
		p-coumaric acid	Phenolic acids	-	[146,147,148]
*Crataegus monogyna* Jacq.	fruits, leaves, flowers	chlorogenic acid	Phenolic acids	-	[148,155,156]
		quinic acid	Phenolic acids	This compound demonstrated neuroprotective and antidepressant effects in LPS-induced neuroinflammation models, ameliorating altered affective and cognitive behaviors. The mechanism of action is mainly associated with anti-inflammatory and antioxidant effects, by inhibiting proinflammatory mediators and oxidative stress at the hippocampal level. Specifically, quinic acid acts by modulating the MAPK/ERK signaling pathway, reducing astrocyte activation and nitrite release, which leads to a decrease in neuroinflammation. Thus, its effect is the result of regulating glial cell activation and neuronal protection under conditions of inflammatory stress.	[157]
		ferulic acid	Phenolic acids	-	[148,155,156]
		caffeic acid	phenolic acids	-	[148,155,156]
		oleanolic acid	Pentacyclic triterpenic acids	-	[148,155,156]
		ursolic acid	Pentacyclic triterpenic acids	-	[148,155,156]
		crataegolic acid	Pentacyclic triterpenic acids	It demonstrated neuroprotective effects in paraquat-induced oxidative stress models. The mechanism of action mainly involves the inhibition of neuronal apoptosis, by reducing ROS formation, decreasing caspase-3 activity, and preventing Ca^2+^ overload. In addition, crataegolic acid modulates apoptotic proteins, by increasing the expression of Bcl-2 (antiapoptotic) and decreasing Bax (proapoptotic). Thus, its effect is the result of an antioxidant and antiapoptotic action, which contributes to neuronal protection.	[158]
		betulinic acid	Pentacyclic triterpenic acids	It exhibits neuroprotective effects by improving motor and cognitive dysfunctions. The mechanism of action is mainly attributed to antioxidant activity, by reducing lipid peroxidation (↓ MDA) and increasing antioxidant enzymes (↑ SOD, ↑ GPx), which limits neuronal oxidative stress. In addition, betulinic acid contributes to increasing neuroplasticity by stimulating BDNF and exerts an anti-inflammatory effect (↓ IL-10), supporting neuronal protection. Thus, its action is based on neutralizing free radicals, reducing inflammation and supporting neuronal function.	[159]
		β-sitosterol	Sterols	Both intraperitoneal and oral administration to mice have been shown to have anxiolytic effects. By injection into mice, β-sitosterol can reduce the effects of restraint stress, contextual fear memory, and c-Fos activation in the prefrontal cortex and dentate gyrus.	[160]
		catechin	Flavonoids	-	[148,155,156]
		(–)-epicatechin	Flavonoids	It exhibited antidepressant effects in a chronic stress model by reducing anhedonia and anxious behaviors. The mechanism of action is mainly associated with the modulation of KAT aminotransferase, an enzyme involved in the metabolism of the kynurenine pathway. Through this action, epicatechin contributes to the regulation of tryptophan metabolism and the neuroactive balance between neurotoxic and neuroprotective metabolites, promoting resilience to stress. Thus, its antidepressant effect is linked to the optimization of the kynurenine pathway and neurochemical homeostasis.	[161]
		rutin	Flavonoids	-	[148,155,156]
		hesperidin	Flavonoids	-	[148,155,156]
		astragalin	Flavonoids	-	[148,155,156]
		procyanidin	Flavonoids	This compound demonstrated antidepressant effects in the LPS-induced neuroinflammation model, reducing depressive behaviors in mice. The mechanism of action is mainly associated with anti-inflammatory effects, by inhibiting the NF-κB pathway, which leads to a decrease in the expression of proinflammatory cytokines and the enzymes iNOS and COX-2 in the hippocampus, prefrontal cortex, and amygdala. Through these actions, proanthocyanidin reduces neuroinflammation and contributes to the improvement of depressive symptoms.	[162]
		hyperoside	Flavonoids	-	[148,155,156]
		vitexin	Flavonoids	In behavioral models, by reducing immobility time in mice, it may exhibit antidepressant effects. The mechanism of action is mainly associated with the modulation of monoaminergic systems, by increasing the level of catecholamines at the synaptic level. The effect involves interactions with dopaminergic receptors (D1, D2, D3) and α2 adrenergic and serotonergic 5-HT1A, an aspect supported by the blocking of the effect in the presence of specific antagonists. Thus, vitexin acts by potentiating dopaminergic, noradrenergic, and serotonergic neurotransmission, contributing to its antidepressant effect.	[163]
		orientin	Flavonoids	It demonstrated antidepressant effects in a chronic stress model, improving depressive behaviors and associated dysfunctions in mice. The mechanism of action mainly involves reduction in oxidative stress by restoring redox balance at the cerebral level, associated with the increase in monoaminergic neurotransmission (serotonin and noradrenaline in the hippocampus and prefrontal cortex). In addition, orientin stimulates neuroplasticity by increasing the expression of BDNF and synaptic proteins (synaptophysin and PSD-95). Thus, the antidepressant effect is the result of an integrated antioxidant, neuromodulatory. and neurotrophic action.	[164]
		apigenin	Flavonoids	-	[148,155,156]
		eriodictyol	Flavonoids	According to studies, it has antidepressant effects and improves cognitive deficits in models of chronic stress and neuroinflammation in rats. The mechanism of action is mainly associated with the modulation of the TRPV1 receptor, highlighted by the synergistic effect with the antagonist capsazepine, suggesting a functional inhibition of this pathway involved in the stress response. In addition, its anti-inflammatory and antioxidant properties contribute to the reduction in neuroinflammation and oxidative stress, supporting neuronal protection.	[165]
		kaempferol	Flavonoids	-	[148,155,156]

Legend: chronic unpredictable mild stress—CUMS; FST—forced swim test; TST—tail suspension test; SST—sucrose splash test; GRg1—Ginsenoside Rg1; GO-PEG-BO—novel-brain-targeted drug delivery system based on borneol-modified PEGylated graphene oxide; LPS—lipopolysaccharide; TRPV1—transient receptor potential vanilloid 1; KAT—kynurenine aminotransferase; ROS—reactive oxygen species; MAO-A—monoamine oxidase A; tyrosine hydroxylase—TH; CRS—chronic restraint stress; CRF—corticotropin releasing factor; BDNF—Brain-Derived Neurotrophic Factor.

**Table 4:** Central and peripheral/systemic toxicity profiles of selected medicinal plants used in anxiety and depression.

Plant	Central Toxicity	Peripheral/Systemic Toxicity	Toxicity Observations	Refs.
*Matricaria chamomilla* L.	Vomiting, immediate hypersensitivity reactions, severe anaphylaxis.	Increased risk of bleeding through interference with coagulation; potential teratogenicity; disruption of the menstrual cycle; reduction in follicular function and development; risk of premature birth; possible oxytocic and abortifacient effect; contact dermatitis and allergic reactions.	Toxicity is insufficiently investigated; possible genotoxic risk.	[85,174,175,176,177,178,179]
*Galium odoratum* (L.) Scop.	Dizziness, fatigue, headaches, seizures, paralysis.	Vomiting, colic, respiratory difficulties; potentially hepatotoxic and possibly carcinogenic.	Human toxicity is poorly documented; the risk is mostly correlated with coumarin content.	[57,75,184,185,186,197,198,199,200,201,202,203,247]
*Melissa officinalis* L.	Dizziness, agitation, headache, decreased alertness, EEG changes at high doses, dose-dependent neurotoxicity	Vomiting, wheezing, abdominal pain, nausea, appetite stimulation, increased intraocular pressure, palpitations, influence on thyroid hormone secretion; with topical use: erythema, contact dermatitis, burning sensation, paresthesia, residual hyperpigmentation, irritation; with high doses/prolonged administration: liver and kidney damage.	Generally considered safe and well tolerated, but high doses and prolonged administration can induce liver, kidney, and neuronal toxicity.	
*Leonurus cardiaca* L.	Excessive sedation when combined with benzodiazepines or other CNS depressants.	Increased risk of bleeding due to antiplatelet effect; uterotonic/emmenagogue activity with risk of uterine bleeding, diarrhea and gastric irritation; contact dermatitis and photosensitivity.	Well tolerated at therapeutic doses; low oral toxicity in animal studies.	[126,204,208,209,210,211,212,213]
*Hypericum perforatum* L.	Dizziness, headache, fatigue, agitation, may induce manic episodes in susceptible patients.	Photosensitivity, erythema, pruritus, skin reactions to UV; nausea, abdominal discomfort, constipation, diarrhea; liver and kidney damage.	One of the best-studied plants regarding adverse reactions and drug interactions; low acute toxicity profile.	[209,224,225,226,227,228,230]
*Tilia platyphyllos* Scop., *Tilia tomentosa* Mnch., *Tilia cordata* Mill.	Mild-to-moderate sedation, drowsiness, decreased alertness, possible sedative potentiation when combined with CNS depressants.	Mild drops in blood pressure, transient palpitations; mild allergic reactions, skin rash, itching.	Generally considered safe; solid evidence of systemic toxicity in healthy individuals is lacking.	[233,239,240,241,248]
*Crataegus monogyna* Jacq.	Drowsiness, agitation, headache, dizziness, migraine.	Sweating, rash, palpitations, mild gastrointestinal upset.	In therapeutic doses, it generally produces mild side effects; caution is required in patients undergoing cardiovascular treatment.	[156,245,246]

**Table 5:** Pharmacokinetic profile, herb–drug interactions, and clinical implications of selected medicinal plants used in anxiety and depression.

Plant	Pharmacokinetics (Available Data)	Drug–Plant Interactions	Clinical Implications	Reference
*Hypericum perforatum* L.	Strong inductor CYP3A4, CYP2C9, CYP2C19, CYP2B6 and P-gp	Reduces plasma concentrations of cyclosporine, digoxin, oral contraceptives, antiretrovirals; risk of serotonin syndrome with SSRIs	Clinically major interactions; require avoidance or close monitoring	[249]
*Matricaria chamomilla* L.	Limited data; possible CYP3A4 inhibition (in vitro)	Isolated cases of interaction with warfarin; possible additive sedative effect	Weak evidence; caution with anticoagulants and sedatives	[250]
*Melissa officinalis* L.	Insufficient data; possible effect on the GABAergic system	Possible additive effect with sedatives and CNS depressants	Interactions not clinically confirmed; caution recommended	[251]
*Leonurus cardiaca* L.	Pharmacokinetic data absent	Possible additive effect with sedatives, antihypertensives; possible effect on coagulation	Insufficient evidence; clinical caution	[252]
*Tilia cordata* Mill./*T. platyphyllos* Scop.	No relevant pharmacokinetic data	No interactions reported; possible additive sedative effect	Safe traditional use; caution with sedatives	[233]
*Tilia tomentosa* Moench	Very limited data	Possible GABAergic effect → theoretical interactions with sedatives	Lack of clinical data; use with caution	[56]
*Crataegus monogyna* Jacq.	Limited data; does not significantly alter digoxin pharmacokinetics	Possible pharmacodynamic interactions with digoxin, antihypertensives	Recommended monitoring in cardiovascular therapies	[253]
*Galium odoratum* (L.) Scop.	Missing data	No clinical evidence; contains coumarin → theoretical potential	Insufficient evidence; theoretical caution	[254]

**Table 6:** Patents on selected herbs used in the treatment of depression and anxiety.

Plant Species	The Manufactured Product	In Combination with Other Plant Extracts or Active Ingredients	Country	Year	Patent Number	Medicinal Uses	Ref.
*Matricaria chamomilla* L.	Dietary supplement	lemon balm, passio flower	US	2019	US10369182B2	treatment of insomnia, sleep disorders, circadian rhythm disorders, anxiety and stress management	[255]
	Dietary supplement	ashwagandha root, hops flower, passionflower, lemon balm leaf		2019	US10517322B1	supporting sleep and relaxation, reducing stress and anxiety	[256]
	Dietary supplement	passionflower, lemon balm, jujube, catnip, Persian silk tree, hemerocallis fulva var. sempervirens, st. john’s wort, bacopa monnieri, luobuma, reishi mushroom, kava, ashwagandha, skullcap		2020	US2020/0138783 A1	sleep promoter, anxiolytic, regulates sleep–wake cycle	[257]
*Melissa officinalis* L.	Non-alcoholic functional drink	panax ginseng, linden, citrus, crataegus, oats, lavender	EU	1996	EP0501591B1	calming, soothing, tonic and gentle energizing effect	[258]
	Dietary supplement	hops cone extract, lavender flower extract, Passionflower extract, skullcap; deodorized valerian root and willow bark extract	US	2011	USOO7914826B2	supporting sleep, inducing nervous relaxation, reducing minor pain	[259]
	Food supplement	vitamins, minerals, amino acids, enzymes, probiotics, plant extracts	US	2013	US2013 0034530A1	supporting cognitive function, improving memory, increasing concentration, supporting learning, maintaining mental clarity, emotional and behavioral stability, supporting nervous system health	[260]
*Leonurus cardiaca* L.	Food composition	*Mentha arvensis* L.	KR	2020	KR102506064B1	prevention and treatment of cognitive dysfunctions	[261]
	Both a medicine and a biologically active food supplement	single	RU	2015	RU2589507C1	reduction in nervous tension, states of increased nervous excitability, neuroses, insomnia, states of restlessness and irritability, functional disorders of the nervous system, cardiovascular manifestations of nervous origin	[262]
	Drug	valerian and hawthorn	RU	2015	RU2563190C2	sedative and anxiolytic, reducing nervousness and irritability, improving sleep, improving functional disorders of the central nervous system, supporting functional cardiovascular disorders	[263]
	Drug	valerian, hawthorn, mint, peppermint essential oil	RU	2020	RU2737701C1	prophylaxis and treatment of psycho-emotional disorders, anxiety, restlessness, irritability, panic attacks, insomnia, functional disorders of the nervous system, hyperthyroidism accompanied by tachycardia, states of increased nervous excitement and palpitations	[264]
*Hypericum perforatum* L.	Food supplement	chlorella (powder), spirulina, *Humulus lupulus* L., *Matricaria chamomilla* L.	KR	2024	KR102651346B1	reducing mental stress, calming the nervous system, improving sleep (falling asleep faster + sleeping longer), increasing melatonin and serotonin levels, decreasing cortisol levels	[265]
	Pharmaceutical composition	hyperforin (standardized extract, hypericin-free), kavapyrone (from *Piper methysticum* G.Forst.), linalool and linalyl acetate (from *Lavandula angustifolia* Mill.), S-adenosyl-L-methionine	EU	2018	EP3111930B1	mild–moderate depression, recurrent depression, persistent affective disorders, bipolar disorders (as adjuvant)	[266]
	Food supplement	5-methyltetrahydrofolic acid, vitamin B12, betaine anhydrous	US	2006	US7014865B1	Preventing and treating mild-moderate depression	[267]
	Food supplement	ashwagandha, passionflower, skullcap, chamomile	US	2015	US2015 0320814A1	reducing anxiety, relieving depression, improving sleep, inducing relaxation and reducing stress	[268]
*Tilia platyphyllos* Scop., *Tilia tomentosa* Mnch., *Tilia cordata* Mill.	Medicinal floral tea	*Nelumbo nucifera* Gaertn., *Lonicera japonica* Thunb., *Lavandula angustifolia* Mill., *Osmanthus fragrans* Lour., *Lilium brownii* F.E.Br., *Ziziphus jujuba var. spinosa* (Bunge) Hu ex H.F.Chow, *Albizia julibrissin* Durazz., *Lycium barbarum* L., *Bambusa vulgaris* Schrad. ex J.C.Wendl., *Cinnamomum cassia* (L.) J.Presl, *Glycyrrhiza uralensis* Fisch.	CN	2015	CN104886311A	Insomnia, difficulty falling asleep, shallow/restless sleep, nervous agitation, mental stress, emotional tension, mental fatigue	[269]
	Food supplement (syrup)	*Melissa officinalis* L., *Matricaria recutita* L.	FR	2017	FR3040628A1	induces relaxation, facilitating falling asleep, improving sleep quality (children and adults)	[270]
	Herbal tea (ORIGITEA^®^)	*Mentha × piperita, Leonurus cardiaca* L., *Calendula officinalis* L., *Humulus lupulus* L., *Coriandrum sativum* L., *Avena sativa* L.	RU	2011	RU2435453C1	stress reduction, calming the nervous system, reducing nervous excitability, difficulty falling asleep, restless sleep, mild insomnia, supporting the functioning of organs and systems, eliminating toxins, improving general condition	[271]
	Food supplement (jelly)	*Valeriana officinalis* L., *Citrus x aurantium* L., *Passiflora incarnata* L.	-	2025	WO2025137727A1	improving sleep quality, facilitating falling asleep, reducing stress, calming the nervous system, reducing agitation before bedtime	[272]
*Crataegus monogyna* Jacq.	Food supplement	*Polygonatum sibiricum* F.Delaroche, *Lycium barbarum* L., Lilium spp., *Rubus chingii* Hu, *Coix lacryma-jobi* L., *Dioscorea opposita* L., *Platycodon grandiflorus* (Jacq.) A.DC., *Ziziphus jujuba var. spinosa* (Bunge) Hu ex H.F.Chow, *Citrus reticulata* Blanco, *Morus alba* L., *Lonicera japonica* Thunb., *Taraxacum mongolicum* Hand.-Mazz., *Nelumbo nucifera* Gaertn., *Cassia obtusifolia* L., *Cinnamomum cassia* Nees ex Blume, *Zingiber officinale* Roscoe	CN	2018	CN108926702A	reduces chronic fatigue and stress, improves sleep quality and mental state, supports digestion, increases immunity, combats irritability and anxiety	[273]
	Drug	*Bupleurum chinense* French., *Citrus x aurantium* L., *Aucklandia lappa* DC., *Citrus reticulata* Blanco, *Pinellia ternata* (Thunb.) Makino, *Taraxacum mongolicum* Hand.-Mazz., *Areca catechu* L., *Paederia foetida* L., *Codonopsis pilosula* (Franch.) Nannf., *Corydalis yanhusuo* (Y.H.Chou & Chun C.Hsu) W.T.Wang ex Z.Y.Su & C.Y.Wu	CN	2025	CN120078853A	Treating depression	[274]

**Table 7:** Herbal preparations available as OTC or dietary supplements used in depression and anxiety, available on the Romanian market.

Trade Name	Manufacturer and Product Code/Marketing Authorization Holder	Product Type	Active Substances	Mode of Administration	Therapeutic Activity
Bellflower, ginseng	Pro Natura [6420488003418]	Food supplement	St. John’s wort aerial parts powder, white ginseng root powder	2–3 capsules/day	anxiolytic, sedative
Bellflower	Bronson [716563111984]	Food supplement	Bellflower extract	1 capsule × 2/day	improving mood and increasing energy levels
Bellflower	Hypericum [6422572000808]	Food supplement	100% natural St. John’s wort extract	adults: 1–2 capsules × 3/day children ≤ 8 years: 3 capsules/day	anxiolytic, mild antidepressant
Distonoplant Forte N171	Fares [5941141014752]	Food supplement	*Galium odoratum* (L.) Scop., *Rhodiola rosea* L., *Withaniae somniferae radix*, *Convolvulus prostrates* Forssk., *Passiflorae herba*, *Lippiae citriodorae aetheroleum*, *Aurantii amari folii aetheroleum*	1–2 capsules × 2 times/day	Anxiolytic antidepressant
Emocalm	Dacia Plant [6421930322743]	Food supplement	*Melissa officinalis* L., *Hypericum perforatum* L., *Lavandula augustifolia* Mill., *Valeriana officinalis* L.	adults and young people over 15 years: 1 drops × 3–5/day children between 5 and 15 years: 1 drops × 3/day	optimal relaxation, supporting mental and physical relaxation, supporting calm in case of irritability, maintaining emotional balance
St. John’s wort and magnesium	DVR Pharm [6426070646384]	Food supplement	*Hypericum perforatum* L., marine magnesium, natural extract from seawater with 55% pure magnesium	1–2 capsules × 2/day	sleep disorders (difficulty falling asleep, frequent nighttime awakenings, restless sleep) anxiety, anxiety–depressive syndrome panic attacks
Emocalm with EPA from algae	Dacia Plant [6421930323627]	Food supplement	Algae extract (Omega 3 + EPA + DHA), *Crataegus monogyna* Jacq,, *Leonurus cardiaca* L., *Capsella bursa-pastoris* Medik, *Terminalia arjuna* (Roxb. exDC.) Wight & Arn., *Lavandula angustifolia* Mill essential oil	adolescents over 12 years: 1 tablet/day adults: 1 tablet × 2/day	nervous tension, restlessness, irritability, fluctuating emotional states, prolonged stress
Emocalm with valerian	Dacia Plant [6421930300284]	Food supplement	Lemon balm, St. John’s wort, lavender, valerian, orange essential oil	adults and young people over 15 years: 1 tablet/day children between 5 and 15 years: 1/2 tablet per day	harmonization of psycho-emotional state, support of optimal calm and relaxation
Standardized St. John’s wort extract 300 mg	General Nutrition Corporation 048107127374	Food supplement	Standardized St. John’s wort extract (0.3% hypericin)	1 capsule × 3/day	calming, antidepressant and relaxing on the nervous system
ANTISTRES	Fares 5941141006429	Food supplement	*Griffonia simplicifolia* (Vahl ex DC.) Baill., ginseng root, St. John’s wort, passionflower, marjoram, lavender essential oil, *Lippiae citriodorae aetheroleum, Melissae aetheroleum, Citri bergami aetheroleum*	children 10–16 years: 1 capsule × 2/day children over 16 years and adults: 1 capsule × 3/day	adaptogen, nerve tonic, stimulates attention and concentration, induces a state of calm and relaxation, improves sleep quality
Motherwort	Favisan [6421830005739]	Food supplement	*Leonurus cardiaca* L.	1 capsule × 3/day	adjuvant in depression, anxiety, tachycardia, cramps, dysmenorrhea, menopause, epilepsy, hypertension
Motherwort with mistletoe and	Favisan 6421830002493	Food supplement	*Leonurus cardiaca* L., hawthorn, *Viscum album* L.	1 capsule × 3/day	nervousness, stress, hot flashes, calming suffocation in asthma, in all conditions in which the organs are driven by an excitation
Maxi Mag Cardio	Zdrovit 5906204017750	Food supplement	Magnesium, hawthorn extract, Leonurus cardiaca extract and B6 vitamin	1 tablet/day	calming, relaxing, maintaining mental health
Q Tensio with hawthorn and motherwort	Kotys 6426831000738	Food supplement	*Leonurus cardiaca* L., Marine magnesium naturally extracted from seawater, *Crataegus oxyacantha* Walter, *Olea europaea* L., *Arctium lappa* L., *Equisetum arvense* L, *Valeriana officinalis* L.	1 capsule × 3/day	antistress, sedative, anxiolytic, maintaining blood pressure within optimal parameters
Linden	Pro Natura 6420488008451	Food supplement	Flower from *Tilia cordata* Mill	1 capsule × 3/day	sedative, antispasmodic, reducing anxiety and stress
Remotiv	EWOPHARMA, SPOL. S.R.O. W70048001	OTC	Hyperici herba	adults over 18 years: 1 tablet × 2/day	mild depressive disorders

## Data Availability

No new data were created or analyzed in this study.

## Supplementary Materials

The following supporting information can be downloaded at https://www.mdpi.com/article/10.3390/biomedicines14051019/s1, Table S1: Data on the location of areas and geographical coordinates. Figure S1: Distribution of plants according to Romania’s landforms.
