# Supplementary material for: Wild Flora Species from Romania with Anxiolytic and Antidepressant Potential: A Global Perspective—Narrative Review
Source: Biomedicines. 2026 Apr 30;14(5):1019. doi: 10.3390/biomedicines14051019 (PMC13204971; doi:10.3390/biomedicines14051019)
Supplement: Supplementary file 1 [file biomedicines-14-01019-s001.zip › biomedicines-4230753-supplementary.pdf]

Table S1. Data on the location of areas and geographical coordinates

Plant species	Counties	GPS coordinates	Altitude	Data collection date
Matricaria chamomilla L.	Timis	45°48'20.39"N 21°15'53.48"E	96.33 m	08.05.2025
	Brasov	46°02'12.79"N 25°08'09.01"E	497.38 m	22.07.2023
	Ilfov	44°29'04.09"N 26°03'24.02"E	90.12 m	13.05.2024
	Vaslui	46°17'57.14"N 27°44'13.82"E	67.28 m	20.05.2021
	Dambovita	44°45'47.86"N 25°29'37.36"E	206.93 m	01.06.2025
	Salaj	47°15'46.45"N 22°57'39.12"E	191.6 m	08.05.2025
	Teleorman	43°39'46.39"N 25°23'46.44"E	28.37	19.06.2016
	Mures	46°27'48.25"N 24°19'27.84"E	287.96 m	24.05.2025
	Ialomita	44°41'28.14"N 26°26'51.02"E	74,22 m	27.04.2024
	Tulcea	45°10'17.82"N 28°47'29.19"E	53,42	05.06.2017
	Cluj	46°35'19.38"N 23°47'21.17"E	350 m	07.06.2025
	Neamt	46°51'24.83"N 26°49'24.95"E	222.09 m	18.05.2025
Galium odoratum (L.) Scop.	Brasov	45°28'27.32"N 25°17'01.23"E	1.042,22 m	03.07.2022
	Bihor	46°30'33.57"N 22°32'46.76"E	486,48 m	23.07.2022
	Neamt	46°59'32.44"N 25°56'06.26"E	1.017,91 m	18.07.2025
	Mures	46°12'31.18"N 24°46'26.33"E	457,39 m	29.04.2025
	Cluj	46°47'41.63"N 23°24'23.94"E	510,16 m	30.04.2025
	Maramures	47°47'46.89"N 23°56'10.09"E	440,49 m	13.05.2025
	Prahova	45°21'58.20"N 25°31'38.34"E	1.171,76 m	27.05.2025
	Valcea	45°17'23.18"N 24°19'58.33"E	519 m	13.05.2025
	Caras-Severin	45°13'32.93"N 22°28'42.89"E	847,98 m	21.06.2025
	Sibiu	45°48'51.80"N 24°25'43.27"E	510,82 m	14.08.2025
	Harghita	46°22'29.10"N 25°56'21.88"E	941,03 m	25.10.2025
	Iasi	47°00'51.91"N 27°34'25.44"E	285,82 m	02.11.2022

	Covasna	46°07'17.64"N 25°53'04.40"E	1.066,2 m	09.11.2025
	Botosani	48°03'31.83"N 26°23'38.72"E	189,35 m	14.04.2024
	Tulcea	45°13'04.04"N 28°18'09.94"E	131,7 m	24.04.2024
	Gorj	45°12'17.86"N 23°47'01.70"E	983,68 m	14.05.2024
	Alba	46°30'05.04"N 23°25'10.94"E	697,81 m	01.06.2024
	Ilfov	44°30'49.21"N 26°05'26.21"E	90,23 m	06.10.2024
	Bacau	46°63'40.64"N, 26°77'64.17"E	400,4 m	08.06.2023
	Suceava	47°51'14.39"N 25°45'54.78"E	488,75 m	15.05.2022
	Dambovita	45°24'42.95"N 25°38'79.28"E	1192,15 m	16.05.2022
	Hunedoara	45°17'23.52"N 22°59'47.14"E	1.056,5 m	15.09.2022
	Giurgiu	44°31'59.89"N 25°52'04.95"E	134,7 m	16.05.2020
Melissa officinalis L.	Ilfov	44°30'54.00"N 26°02'59.99"E	92,25 m	21.06.2025
	Olt	44°26'37.56"N 24°21'07.74"E	157,71 m	08.06.2025
	Giurgiu	44°08'13.65"N 26°08'47.71"E	88 m	28.06.2025
	Constanta	44°04'05.84"N 27°45'07.14"E	107,84 m	14.07.2025
	Cluj	46°34'51.75"N 23°43'17.09"E	438,51 m	21.07.2024
	Caras-Severin	44°54'23.54"N 22°25'55.14"E	195,19 m	16.08.2025
	Valcea	45°17'59.99"N 24°17'59.99"E	657,08 m	04.10.2025
	Arad	46°06'00.00"N 21°48'00.00"E	146,06 m	19.03.2025
	Arges	45°15'00.00"N 24°38'59.99"E	507,15 m	30.03.2024
	Galati	45°36'00.00"N 27°53'59.99"E	123,64 m	16.05.2024
	Harghita	46°24'40.95"N 25°46'56.04"E	670,98 m	18.05.2024
	Dambovita	44°56'35.85"N 25°28'36.41"E	277,79 m	04.07.2024
	Mehedinti	44°41'59.99"N 22°56'59.99"E	175,06 m	10.09.2024
	Iasi	47°10'01.48"N 27°34'36.63"E	64,23 m	11.05.2023

Brasov	45°39'26.85"N 25°34'55.18"E	555,37 m	06.06.2023
Calarasi	44°25'29.83"N 27°00'38.82"E	47,14 m	26.06.2023
Hunedoara	45°44'52.05"N 22°54'18.96"E	254,04 m	24.04.2022
Ialomita	44°43'36.00"N 27°25'13.00"E	26,58 m	24.04.2022
Sibiu	45°42'24.36"N 24°06'12.57"E	558,79 m	27.04.2022
Mures	46°35'01.24"N 24°28'48.11"E	334,97 m	30.04.2022
Botosani	47°41'59.99"N 26°47'59.99"E	112,72 m	26.04.2022
Suceava	47°32'59.99"N 25°36'00.00"E	1.052,88 m	30.04.2022
Vaslui	46°41'59.99"N 27°53'59.99"E	147,96 m	30.04.2022
Harghita	46°24'40.95"N 25°46'55.42"E	670,86 m	21.05.2022
Bacau	46°30'00.00"N 26°11'59.99"E	585,63 m	29.05.2022
Neamt	46°47'56.05"N 26°57'17.53"E	190,77 m	03.05.2022
Telorman	43°47'59.99"N 25°23'59.99"E	84 m	15.05.2022
Timis	45°44'19.45"N 21°20'10.29"E	92 m	15.05.2022
Braila	45°17'59.99"N 27°53'59.99"E	15,27 m	19.05.2022
Bihor	47°02'19.30"N 21°58'57.91"E	136,23 m	21.05.2022
Prahova	45°00'52.93"N 26°04'01.70"E	272,69 m	15.05.2022
Salaj	47°11'59.99"N 23°11'59.99"E	229,06 m	15.05.2022
Satu Mare	47°47'59.99"N 23°24'43.19"E	329,4 m	21.05.2021
Maramures	47°45'00.00"N 24°00'00.00"E	715,63 m	06.05.2021
Bistrita-Nasaud	47°15'00.00"N 24°23'59.99"E	451,38 m	10.05.2021
Tulcea	45°15'00.00"N 28°35'59.99"E	3,65 m	17.04.2021
Buzau	45°17'59.99"N 26°41'59.99"E	252,93 m	11.08.2022
Vrancea	45°47'59.99"N 27°00'00.00"E	495,37 m	17.07.2022
Covasna	45°47'59.99"N 26°11'59.99"E	945,42 m	08.07.2022

Leonurus cardiaca L.	Alba	46°30'00.00"N 23°18'00.00"E	1.238,98 m	11.06.2022
	Gorj	45°06'00.00"N 23°15'04.31"E	268,01 m	03.06.2022
	Dolj	44°21'00.00"N 23°39'28.79"E	117,28 m	22.05.2022
	Mehedinti	44°34'15.13"N 22°03'09.44"E	532,3 m	10.03.2026
	Caras-Severin	44°54'05.20"N 21°43'22.00"E	153,77 m	26.06.2025
	Cluj	46°46'44.82"N 23°43'11.40"E	315,83 m	20.06.2025
	Constanta	44°04'03.68"N 27°40'22.67"E	145,58 m	16.06.2025
	Ilfov	44°30'48.80"N 25°59'36.93"E	103,04 m	09.04.2024
	Suceava	47°51'50.39"N 26°14'30.59"E	290,12 m	02.04.2023
	Tulcea	45°11'39.55"N 28°26'59.99"E	161,05 m	19.07.2023
	Satu Mare	47°45'18.55"N 23°03'39.43"E	129 m	27.04.2022
	Timis	45°51'26.33"N 20°34'14.70"E	77,67 m	24.04.2022
	Prahova	44°56'53.27"N 26°00'46.25"E	162,87 m	11.04.2022
	Giurgiu	44°07'51.67"N 26°24'06.95"E	75,2 m	24.04.2022
	Calarasi	44°13'39.14"N 26°35'22.02"E	40,27 m	30.04.2022
	Arges	44°51'26.39"N 24°52'56.63"E	261,8 m	02.05.2022
	Maramures	47°28'48.40"N 23°47'56.58"E	377,82 m	18.05.2022
	Harghita	46°07'05.99"N 25°00'42.00"E	499,27 m	29.06.2022
	Botosani	47°56'00.32"N 26°53'59.99"E	110,06 m	18.06.2022
	Vaslui	46°36'00.00"N 27°23'59.99"E	194,55 m	05.07.2022
	Iasi	47°12'33.71"N 27°32'04.21"E	194,93 m	24.04.2021
	Hunedoara	45°53'20.28"N 22°53'43.14"E	306,23 m	12.05.2021
	Bihor	47°32'06.17"N 22°09'12.63"E	131,04 m	09.05.2021
	Dambovita	44°45'52.60"N 25°47'27.86"E	152,6 m	14.05.2021
	Dolj	44°02'44.58"N 24°07'14.12"E	127,35 m	10.05.2021

	Braila	45°53'20.28"N<br>27°53'59.99"E	94,28 m	20.03.2020
	Mures	46°36'01.96"N<br>25°04'00.42"E	464,82 m	29.04.2020+
	Arad	45°53'59.99"N<br>21°53'59.99"E	214,35 m	18.05.2020
	Alba	45°53'20.28"N<br>23°03'00.00"E	491,29 m	20.04.2020
	Teleorman	44°06'00.00"N<br>25°30'00.00"E	88,26 m	01.08.2020
	Gorj	45°11'20.28"N<br>22°53'43.14"E	1.121,18 m	28.05.2020
	Valcea	45°06'00.00"N<br>24°06'00.00"E	417,35 m	18.07.2020
Hypericum perforatum L.	Hunedoara	46°32'26.88"N<br>25°07'37.68"E	491,82 m	22.06.2025
	Mehedinti	44°45'02.64"N<br>22°38'54.92"E	288,51 m	20.06.2025
	Caras-Severin	45°06'59.60"N<br>22°24'57.67"E	914,07 m	21.06.2025
	Cluj	46°52'12.37"N<br>23°52'41.16"E	371,46 m	21.06.2025
	Sibiu	45°56'21.48"N<br>24°33'39.29"E	474,71 m	15.08.2025
	Iasi	47°00'46.72"N<br>27°32'59.94"E	191,44 m	02.11.2025
	Maramures	47°40'12.76"N<br>23°33'17.94"E	445,19 m	15.03.2024
	Harghita	47°40'12.76"N<br>23°33'17.94"E	445,19 m	21.06.2024
	Brasov	45°36'44.07"N<br>25°32'35.19"E	632 m	25.07.2023
	Covasna	45°47'00.90"N<br>26°01'11.99"E	582,23 m	5.07.2023
	Vrancea	46°05'46.63"N<br>27°13'40.87"E	86,79 m	08.08.2023
	Constanta	44°33'08.18"N<br>28°45'47.19"E	0,07 m	23.06.2022
	Alba	46°07'01.99"N<br>23°35'57.98"E	371,25 m	14.07.2022
	Neamt	46°57'48.90"N<br>26°34'29.58"E	341,2 m	28.06.202
	Salaj	46°54'58.00"N<br>23°11'56.46"E	369,22 m	28.06.2019
	Dambovita	44°32'01.56"N<br>25°31'52.77"E	138 m	11.07.2019
	Prahova	45°14'44.78"N<br>25°37'01.83"E	736,81 m	06.07.2019
	Suceava	47°40'51.81"N<br>26°17'54.88"E	365,71 m	26.06.2018

	Giurgiu	44°14'56.65"N 25°48'51.64"E	77,08 m	15.06.2017
	Tulcea	45°15'25.99"N 28°21'00.00"E	135,01 m	28.06.2017
Tilia platyphyllos Scop.	Ilfov	44°26'58.82"N 26°06'12.76"E	81,6 m	03.02.2026
	Dolj	44°18'05.29"N 23°47'58.35"E	79,71 m	06.05.2025
	Arges	44°50'31.39"N 24°53'22.93"E	293,88 m	29.05.2024
	Harghita	46°43'28.97"N 25°36'17.02"E	815,24 m	03.07.2022
	Prahova	45°04'11.58"N 26°00'33.79"E	228,26 m	02.06.2019
	Caras-Severin	44°52'51.52"N 22°25'49.40"E	790,88 m	01.10.2019
	Hunedoara	45°36'51.85"N 22°58'23.47"E	358,89 m	26.04.2018
Tilia cordata Mill.	Iași	47°00'45.94"N 27°34'09.78"E	250,58 m	02.11.2025
	Ilfov	44°25'35.90"N 26°09'39.93"E	73,15 m	03.05.2024
	Tulcea	44°51'43.54"N 28°41'29.71"E	131,25 m	12.03.2022
	Bihor	46°52'41.00"N 22°42'51.85"E	601,04 m	14.08.2022
	Caras-Severin	44°52'51.52"N 22°25'49.40"E	790,88 m	01.10.2019
	Cluj	46°46'47.84"N 23°36'24.69"E	333,72 m	30.08.2021
	Mureș	46°57'01.45"N 25°15'26.81"E	638,33 m	17.08.2021
	Harghita	46°21'38.52"N 25°48'03.59"E	668,09 m	26.07.2021
	Mehedinti	44°38'12.83"N 22°39'34.91"E	78,5 m	03.06.2021
	Sibiu	45°45'25.81"N 24°07'26.81"E	479,95 m	28.05.2025
	Suceava	47°38'32.35"N 26°16'11.40"E	346,97 m	11.06.2024
	Brasov	45°38'21.95"N 25°35'02.74"E	595,29 m	26.04.2023
	Vrancea	45°41'56.48"N 27°10'29.48"E	56,73 m	17.09.2023
Tilia tomentosa Mnch.	Ilfov	44°27'12.08"N 26°00'37.06"E	93,53 m	15.05.2025
	Constanta	44°05'16.93"N 27°44'59.80"E	72,81 m	28.05.2025
	Caras-Severin	44°54'12.13"N 21°43'04.88"E	231,12 m	26.06.2025

	Bihor	46°54'51.60"N 22°36'41.16"E	478,3 m	20.06.2025
	Tulcea	45°13'05.77"N 28°18'28.32"E	115,44 m	24.04.2024
	Timis	45°43'22.60"N 21°10'27.19"E	88,22 m	26.06.2024
	Olt	44°30'22.02"N 24°01'24.30"E	166,9 m	19.07.2024
	Iasi	47°10'42.83"N 27°33'59.94"E	120,65 m	15.08.2024
	Galati	45°49'23.81"N 27°46'09.10"E	198,18 m	18.09.2023
	Calarasi	44°34'14.99"N 27°21'53.99"E	22,81 m	24.04.2022
	Covasna	45°55'58.24"N 26°02'59.99"E	547,97 m	08.05.2022
	Sibiu	45°38'38.14"N 24°16'51.18"E	526,87 m	16.07.2022
	Mehedinti	44°48'06.78"N 22°58'04.03"E	180,73 m	07.07.2020
Crataegus monogyna Jacq.	Arad	46°09'35.27"N 20°33'47.30"E	94,1 m	03.04.2025
	Galati	45°26'36.09"N 27°45'04.11"E	11,76 m	11.04.2025
	Vrancea	45°26'36.09"N 27°45'04.11"E	11,76 m	04.05.2025
	Mehedinti	45°03'36.71"N 22°38'05.35"E	815,13 m	20.06.2025
	Cluj	46°47'04.74"N 23°29'53.58"E	529,21 m	07.04.2026
	Tulcea	45°01'44.93"N 29°24'23.53"E	3,95 m	11.09.2025
	Ialomita	44°33'51.38"N 27°30'44.86"E	18,57 m	24.09.2025
	Giurgiu	44°07'32.93"N 26°11'09.97"E	89,45 m	28.05.2025
	Bihor	47°02'41.82"N 21°55'10.66"E	124,68 m	10.10.2025
	Alba	45°51'50.69"N 23°02'31.63"E	264,11 m	12.10.2025
	Constanta	43°57'34.48"N 28°00'32.46"E	137,58 m	23.22.2025
	Buzau	45°11'29.78"N 26°11'23.17"E	255,14 m	13.12.2025
	Vaslui	46°08'26.84"N 27°34'51.25"E	62,4 m	26.04.2024
	Timis	45°49'48.91"N 21°11'15.82"E	90 m	29.04.2024
	Brasov	45°42'37.83"N 25°41'15.18"E	527,35 m	28.08.2024

Ilfov	45°42'37.83"N 25°41'15.18"E	527,35 m	16.09.2024
Valcea	45°07'30.53"N 24°10'17.29"E	362,28 m	03.05.2023
Hunedoara	45°35'05.28"N 22°45'44.03"E	489,78 m	11.05.2023
Iasi	47°04'30.11"N 27°39'39.50"E	352,08 m	15.09.2023
Mures	46°14'49.53"N 24°29'41.25"E	478,78 m	02.01.2022
Sibiu	45°38'38.14"N 24°16'51.18"E	526,87 m	16.07.2022
Harghita	46°32'26.88"N 25°07'37.68"E	491,82 m	10.08.2022

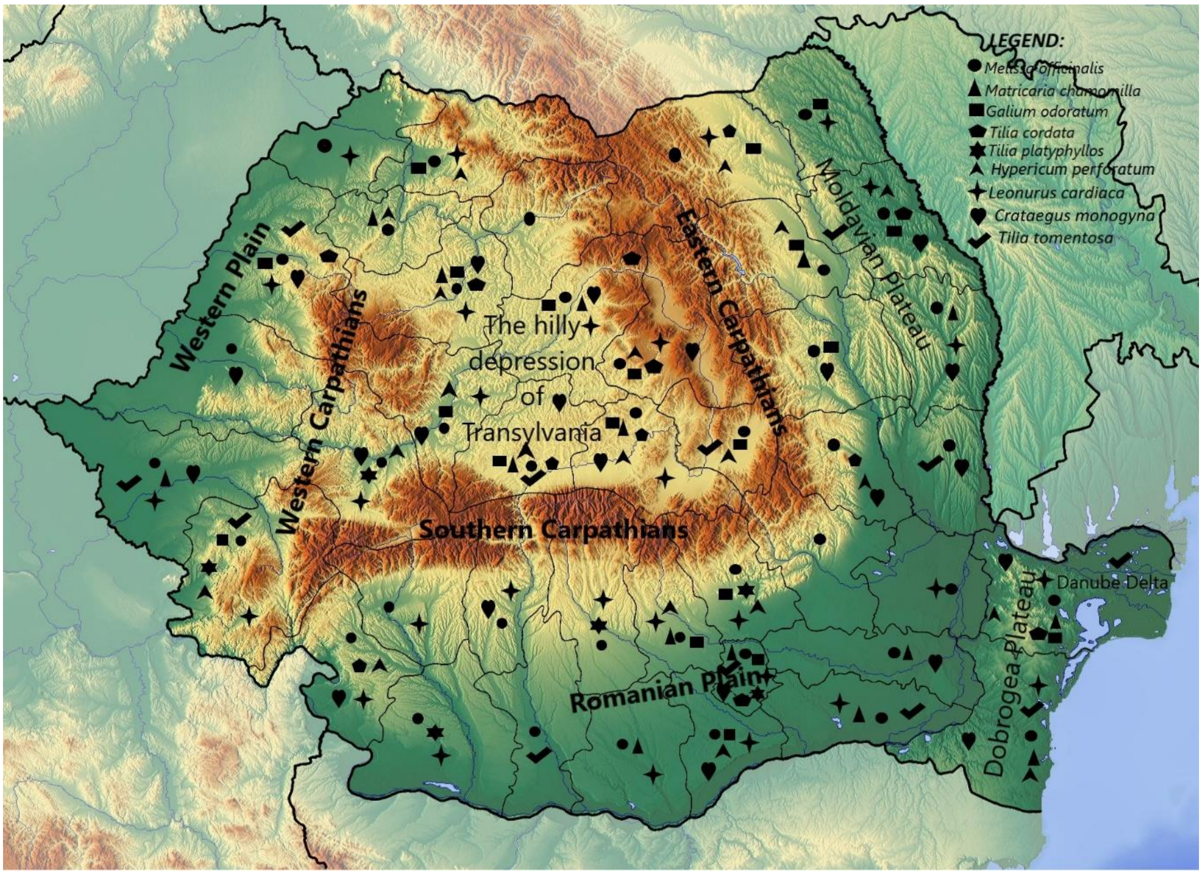

Figure S1. Distribution of plants according to Romania's landforms
